# Resistance to paclitaxel is associated with a variant of the gene BCL2 in multiple tumor types

**DOI:** 10.1038/s41698-019-0084-3

**Published:** 2019-04-23

**Authors:** Rotem Ben-Hamo, Alona Zilberberg, Helit Cohen, Keren Bahar-Shany, Chaim Wachtel, Jacob Korach, Sarit Aviel-Ronen, Iris Barshack, Danny Barash, Keren Levanon, Sol Efroni

**Affiliations:** 1 The Mina and Everard Goodman Faculty of Life Sciences, Bar Ilan University, Ramat-Gan, 52900 Israel; 2 The Broad Institute of Harvard and MIT, Cambridge, MA USA; 3 Department of Molecular Cell Biology, Weizmann Institute of Science, Rehovot, Israel; 4 Sheba Cancer Research Center, Chaim Sheba Medical Center, Ramat-Gan, 52621 Israel; 5 Department of Gynecologic Oncology, Chaim Sheba Medical Center, Ramat-Gan, 52621 Israel; 6 Department of Pathology, Chaim Sheba Medical Center, Ramat-Gan, 52621 Israel; 7 Talpiot Medical Leadership Program, Chaim Sheba Medical Center, Ramat-Gan, 52621 Israel; 8 Sackler Faculty of Medicine, Tel Aviv University, Ramat Aviv, 69978 Israel; 9 Department of Computer Science, Ben Gurion University of the Negev, Beer Sheva, 84105 Israel; 10 The Dr. Pinchas Borenstein Talpiot Medical Leadership Program 2012, Institute of Oncology, Chaim Sheba Medical Center, Ramat-Gan, 52621 Israel

**Keywords:** High-throughput screening, Cancer genomics, Molecular biology

## Abstract

Paclitaxel, the most commonly used form of chemotherapy, is utilized in curative protocols in different types of cancer. The response to treatment differs among patients. Biological interpretation of a mechanism to explain this personalized response is still unavailable. Since paclitaxel is known to target BCL2 and TUBB1, we used pan-cancer genomic data from hundreds of patients to show that a single-nucleotide variant in the BCL2 sequence can predict a patient’s response to paclitaxel. Here, we show a connection between this BCL2 genomic variant, its transcript structure, and protein abundance. We demonstrate these findings in silico, in vitro, in formalin-fixed paraffin-embedded (FFPE) tissue, and in patient lymphocytes. We show that tumors with the specific variant are more resistant to paclitaxel. We also show that tumor and normal cells with the variant express higher levels of BCL2 protein, a phenomenon that we validated in an independent cohort of patients. Our results indicate BCL2 sequence variations as determinants of chemotherapy resistance. The knowledge of individual BCL2 genomic sequences prior to the choice of chemotherapy may improve patient survival. The current work also demonstrates the benefit of community-wide, integrative omics data sources combined with in-lab experimentation and validation sets.

## Introduction

Chemotherapy resistance is a major obstacle for the success of cancer therapy. Clinical drug resistance can be defined as either a lack of reduction in the size of the tumor following chemotherapy or the occurrence of clinical relapse after an initial “positive” response to treatment.^1^

Causing more deaths than any other gynecological cancer, epithelial ovarian cancer had an estimated 22,280 new cases and 15,500 deaths in the United States in 2012.^2^ Ovarian cancer strikes silently, revealing no obvious symptoms until late in its course, leading to late-stage diagnosis.^3^ The recent increase in genome-wide association studies has revealed a substantial contribution of synonymous SNPs to human disease risk and other complex traits.^4,5^ Recent studies have shown the dramatic effect that synonymous SNPs may have on the stability of the mutated mRNA.^6–9^ Griseri et al.^9^ showed that a synonymous polymorphism in TTP affects translation efficiency and response to Herceptin treatment in breast cancer.

The BCL2 protein promotes cell survival by inhibiting programmed cell death or apoptosis.^10,11^ A variety of chemoresistant carcinomas, including ovarian cancer, uterine cancer, breast cancer, and gastric cancer, express BCL2, suggesting that BCL2 may interfere with a variety of cytotoxic agents, including platinum.^12,13^ The most commonly used form of chemotherapy, paclitaxel,^14^ is utilized in curative protocols in different types of cancer, with differing levels of response to treatment. For example, in ovarian cancer,^15^ the current protocol determines that ~40% of patients would respond to first-line treatment of paclitaxel and platinum, while the other 60% continue to undergo additional different chemotherapy cycles with agents, such as tamoxifen, topotecan, doxorubicin, OvaRex, bevacizumab, letrozole, melphalan, gemcitabine, bevacizumab, capecitabine, and vinorelbine.^16–18^

Currently, there are no pretreatment tests that allow for the personalized tailoring of chemotherapy, and the majority of patients receive this treatment with no impact on disease state.^19–21^ Thus, there is an urgent need to uncover patient-specific traits that may assist in choices of therapy, prevent unneeded suffering, and improve survival by directing patients to effective therapy.

In the work presented here, we used pan-cancer genomic data from hundreds of patients to show that a single-nucleotide variant in the BCL2 sequence can predict a patient’s response to paclitaxel, a widely used chemotherapy. Furthermore, we highlight a connection between this BCL2 genomic variant, its transcript structure, and protein abundance. Finally, our results indicate BCL2 sequence variations as determinants of chemotherapy resistance. Taken together, this knowledge, prior to the choice of chemotherapy, may improve patient survival and patient care.

## Results

### A T > C variation at position 21 of the BCL2 sequence predicts response to paclitaxel

To find genetic determinants of ovarian cancer patient resistance to paclitaxel, we studied genomic differences between such patients in the two genes that are targets of paclitaxel, TUBB1, and BCL2.^22–25^ Our goal was to identify somatic or germline variations in the sequences of these two genes in the two clinical groups with the hope that these variations may correlate with therapy resistance.

We looked at all genomic variants in the two genes between patients using whole-exome sequencing data and clinical data from a pan-cancer set of patients who were all treated with paclitaxel as a first-line treatment. This set of patients is available from The Cancer Genome Atlas (TCGA) and includes 370 OV patients, 83 UCEC patients, and 28 HNSC patients.

**Fig. 1:**
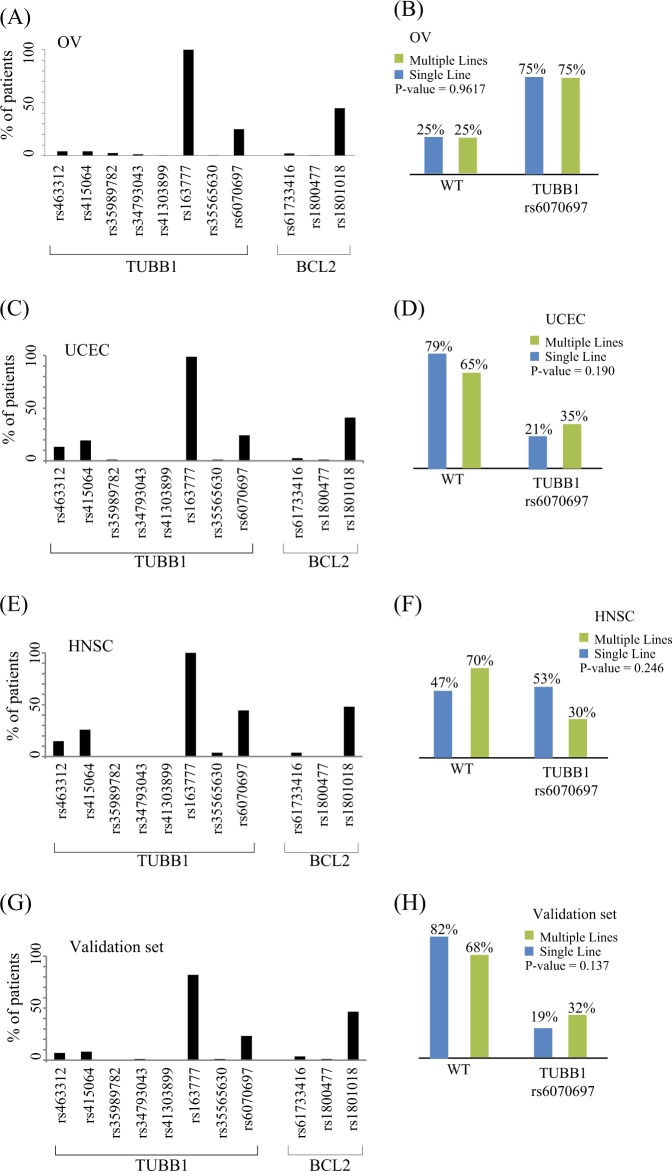
Eleven SNPs were identified within TUBB1 and BCL2. **a** In OV, two SNPs (rs6070697, rs1801018) present a frequency that may be of use for the stratification of patients according to their response status. **b** rs6070697 is of the same frequency in both groups (*t* test *p*-value < 0.96). A study of the polymorphisms of TUBB1 and BCL2 in UCEC shows similar results (**c**) for all polymorphism, while a more careful study (**d**) of rs6070697 again demonstrates that it does not present a significant difference in the two response groups (*t* test *p*-value < 0.19), and a study of HNSC follows these (*t* test *p*-value < 0.25) findings (**e**, **f**), as does a validation set composed of data from all TCGA patients that were treated with a first line of paclitaxel (excluding OV, UCEC, and HSNC) of other types of cancer. This validation set includes sequence data from 86 patients with BLCA, CESC, ESCA, LUAD, LUSC, SKCM, STAD, UCS. Similar to OV, UCEC and HNSC, this combined set does not present any polymorphism with significant association to response (**g**), including the candidate rs6070697 (*t* test *p*-value < 0.13) (**h**)

Combined, TUBB1 and BCL2 have 11 variable loci (Fig. 1b and Fig. S1, Tables S1–S4). These variable regions have all been recorded as single-nucleotide polymorphisms (SNPs). Among these 11 loci, eight showed variation in <4% of patients; one was present in 100% of patients (and 100% of the control population) and one (rs6070697) was distributed equally among the two treatment groups. Therefore, due to a lack of variation, these loci were excluded from further analysis.

However, one variation, the transition from a C to a T at location 21 of BCL2 (+ 21 T > C, Fig. 1b),^26^ showed a significant association with resistance to paclitaxel (Fig. 1c).

This variation is referred to as rs1801018 in the NCBI’s public archive of all short-sequence variations (dbSNP). The variation status matched patient resistance to paclitaxel in a manner that was able to retrospectively predict whether the patient received single or multiple lines of treatment. A total of 202 of 370 OV patients carried a T instead of a C (in one or two alleles). In total, 73% of patients had a T at location 21 and did not respond to Taxol–platinum first-line therapy and eventually received multiple lines of treatment (Fig. 1d, e). In contrast, 74% of the 144 single-line treatment responders (Fig. 1e) presented a C and not a T at location 21. Similar results were found in samples from UCEC patients (Fig. 1f, g), where 70% of single-line responders displayed a C at location 21, while 75% of multiple-line nonresponders displayed a T instead of a C. Similarly, in 27 HNSC patients, 65% of single-line responders displayed a C, while 70% of the multiple-lines group displayed a T (Fig. 1h, 2b). The findings for all variable loci are summarized in Table S5. In the table, we can see that a variation at location 21 is the only variation that presents a meaningful association (a significant *p*-value) with resistance to treatment in each of the sets.

**Fig. 2:**
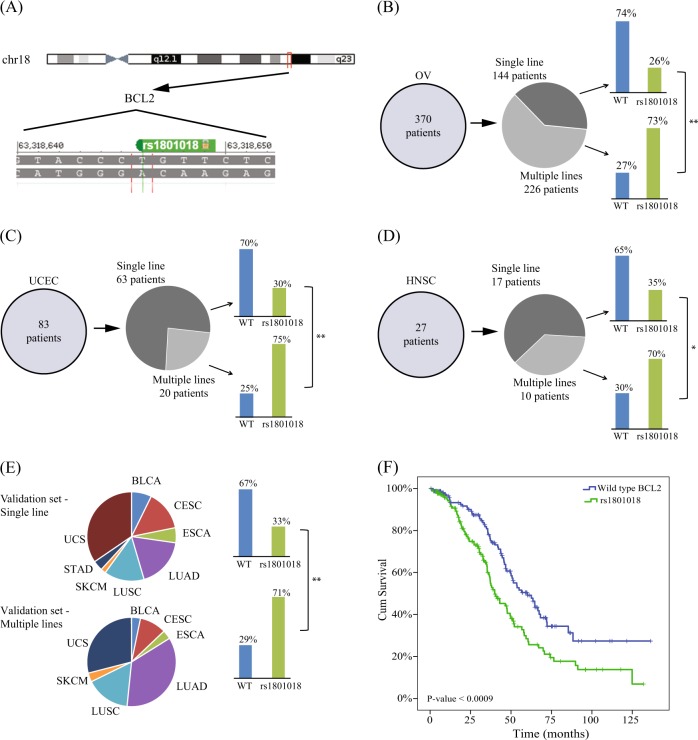
**a** OV, 144 patients have responded well to the first-line treatment, while 226 patients required additional lines of diverse chemotherapies (**b**). rs1801018 status is highly correlated with the affiliation to the first-line group versus the multiple-line group (chi-square *p*-value < 10^−16^). **c** rs1801018 in UCEC (chi-square *p*-value < 3 × 10^−4^). Panel **d** shows the stratification of the variant is consistent in HNSC (*p*-value < 0.05), as well as in the pan-cancer validation (chi-square *p*-value < 6 × 10^−4^) set (**e**). *P*-values were calculated using chi-square test. **f** Kaplan–Meier survival curve of ovarian cancer patients harboring rs1801018 SNO and wild-type BCL2

We therefore hypothesized that the BCL2 variant is a determinant of paclitaxel resistance and that the phenomenon is not specific to a type of cancer. We constructed an independent validation set composed of samples from a collection of 86 patients with eight different cancer types who were treated using paclitaxel as the first line. This validation set presented similar results. While only 33% of patients affiliated with single-line responders presented a T at location 21, 71% of multiple-line nonresponders presented the T variant (Fig. 2b–e). In addition, to further test drug efficacy in association with the presence of the mutation, we performed Kaplan–Meier survival analysis to establish the connection between the presence of the SNP and overall survival in response to paclitaxel–platinum treatment in ovarian cancer patients. Figure 2f shows that this polymorphism significantly (*p* < 0.0009) stratified patient survival in response to paclitaxel. This observation strengthens our hypothesis that the BCL2 polymorphism determines the response to paclitaxel.

### The T >C variant at location 21 of BCL2 changes its RNA secondary structure

Recent findings linking SNPs and cellular function indicate that changes in RNA structural features are caused by synonymous sequence alterations. A synonymous variation sometimes influences transcript stability and translation rate.^27–31^

**Fig. 3:**
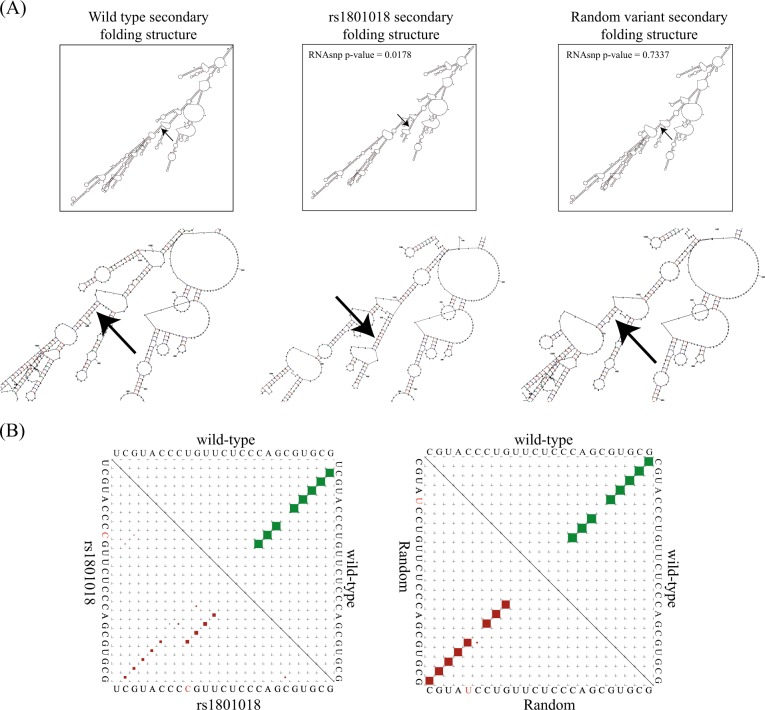
The T > C rs1801018 variant leads to significant structural changes in the mRNA secondary structure, which in turn leads to a more stable transcript and to higher BCL2 protein levels. **a** Predicted secondary structure of BCL2 mRNA. The secondary structure of reference, variant (+21 T > C) or random (+23 C > T) BCL2. The location of the SNP is indicated by an arrow. The analysis was conducted by MFOLD. **b** Base-pair probabilities corresponding to changes in local regions (RNAsnp). A significant change is evident (RNAsnp *p*-value = 0.0178) in the change from reference (green) to the variant (+21 T > C) (red) BCL2 version. No significant changes (RNAsnp *p*-value = 0.7337) were identified for the random variation

Here, we used RNA-folding prediction software^32,33^ to assess structural changes that could result from the C > T change in BCL2. As demonstrated in ref. ^34^, synonymous changes in UTRs that alter the mRNA structural ensemble of the associated genes may be considered candidates for so-called “RiboSNitches”, if the specific variation leads to a structural consequence that results in a disease phenotype (see method evaluation in ref. ^35^). Specifically, we used the tools RNAsnp^36,37^ and RNAplfold^38^ to test the effect of the T at position 21 by comparison with adjacent variations (+ 21 T > C vs. + 23 C > T). The T > C variant (Fig. 3a) presents a lower *p*-value (*p*-value < 0.0178), indicating a structural change that can be traced by comparing dotplots in RNAplfold^38^ (Fig. 3b) of the reference versus the observed change. As an additional control, we also tested a random variation (+ 23 C > T), as shown in Fig. 3b. The higher, nonsignificant *p*-value (0.7337) indicates an insignificant change in structure. Similarly, the reference sequence, compared with the random variation, does not result in a significant change (Fig. 3b).

### Changing location 21 in BCL2 from C to T stabilizes BCL2 RNA secondary structure in vitro

**Fig. 4:**
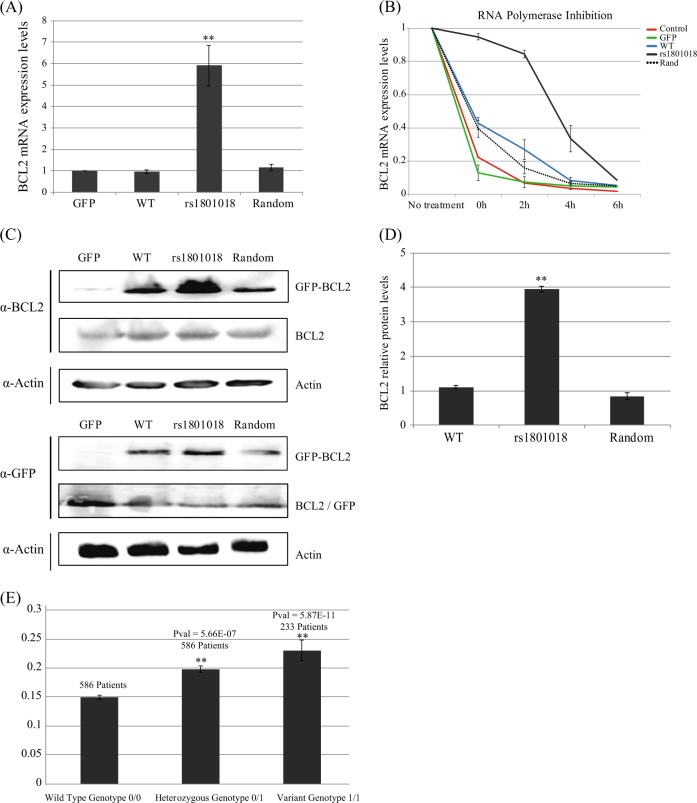
**a** BCL2 mRNA transcript stability determined by specific single-nucleotide variants changes. Relative transcript levels of GFP-BCL2 variants were measured by real-time qPCR (normalized to β-actin). **b** rs1801018 derived BCL2 produces a significantly more stable version of transcript, with an effect lasting 4 to 6 h post induction. **c** Protein levels of the variant BCL2 are higher than reference and random BCL2. All blots derive from the same experiment and were processed in parallel. **d** GFP protein levels were quantified compared with actin using ImageJ. **e** BCL2 RNAseq levels from 1417 patients with seven different types of cancer from, *y*-axis represents averaged FPKM levels, and the *x*-axis represents the SNP genotype. *P*-values were calculated using two-sided *t* test

To experimentally validate these computational results, we studied the effect of sequence modification on transcript stability. Ovarian carcinoma HeyA8 cells were transfected with GFP or one of three GFP-BCL2 variants: reference (+21 T), T > C (+21 C) or random (+23 C > T). HeyA8 cells harbor a homozygous genotype for the reference form of BCL2. At 48 h post transfection, we recovered the total RNA and used qRT-PCR to quantify BCL2 expression levels in all three variants (normalized for GFP as a control for the quality of transfection). The levels of BCL2 in T > C-transfected cells were 5.5-fold higher (*p*-value < 0.004) relative to the reference or the random control (Fig. 4a). Expression levels in the reference and random transcripts were similar to the basal expression levels of BCL2, as represented by GFP-empty vector-transfected cells (Fig. 4a).

These findings rely on the hypothesis that genomic variation alters transcript stability. To measure the stability of the different BCL2 mRNA transcripts, we used the same set of transfected cells and variants described above in the presence of actinomycin D. Incubation with actinomycin D, an RNA polymerase II inhibitor, facilitates tracing kinetics of the different BCL2 transcripts. As demonstrated in Fig. 4b, the transcript of the form of BCL2 that includes the T > C modification with GFP-BCL2 was significantly more stable.

### Changing T to C at location 21 leads to an increase in protein levels in vitro

These findings led us to hypothesize that the + 21 T > C substitution leads, through a more stable transcript, to an increase in BCL2 protein levels. We tested this hypothesis in cell lines, in 1417 patient samples from TCGA, and in an independent cohort of 46 samples (from Sheba Medical Center at Tel-Hashomer, Israel) from ovarian cancer patients. First, we overexpressed the control GFP-empty vector or one of the three BCL2-GFP variants described above. Protein levels were quantified by western blot and are presented in Fig. 4c. The BCL2 protein levels in cells with a C instead of a T at location 21 of BCL2 were fourfold (*p*-value < 1.1e-006) higher than those with the reference sequence.

We then tested for differences in BCL2 expression levels in the RNA-seq data of 1417 pan-cancer samples from TCGA (Fig. 4e and Tables S6–S12). The analysis demonstrated that the expression levels were significantly associated with genotype. Both the heterozygous (598 samples) and homozygous (298 samples) forms of a C at location 21 showed increased protein levels of BCL2 compared to the reference form (586 samples).

**Fig. 5:**
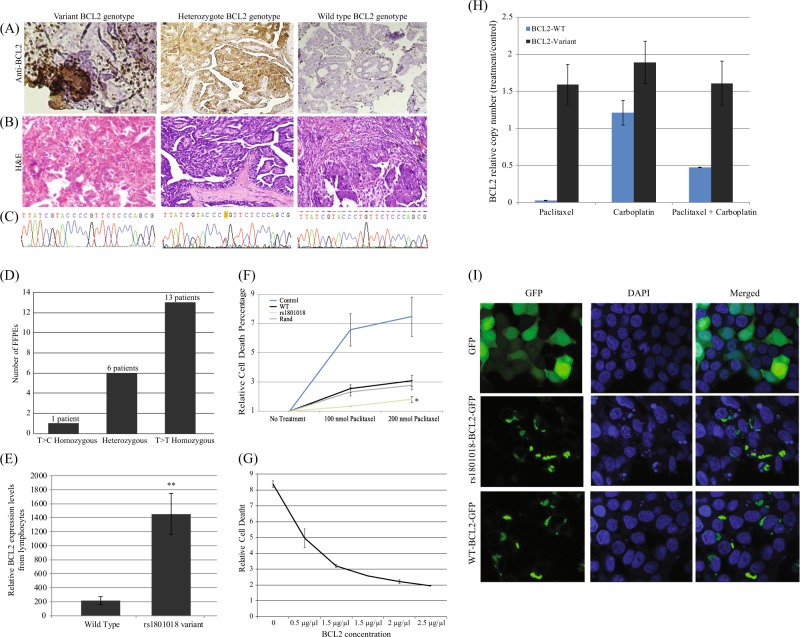
BCL2 genotype correlates with BCL2 positivity in primary tissue from ovarian cancer patients. Representative images of high-grade serous carcinoma stained with BCL2 (**a**) and their matched H&E stains (**b**) and their corresponding Sanger sequencing traces (**c**). FFPE specimens of high-grade serous ovarian tumors from 46 patients were stained with BCL2 and validated (**d**) for their BCL2 genotype. **e** Lymphocytes were extracted from 12 patients (seven patients with a reference sequence and five patients with rs1801018). BCL2 mRNA levels were quantified using qRT-PCR and normalized using endogenous levels of B-actin. **f** Elevated resistance to paclitaxel in the rs1801018 variant (*p*-value < 0.05). While the control strain, expressing the lowest amounts of BCL2, is much more sensitive than all other produced strains, both the reference and the random strains show similar sensitivities, and are significantly more sensitive than the rs1801018 strain. **g** BCL2 gradient assay measures cell sensitivity to paclitaxel in the presence of increasing BCL2 transfection concentrations, y-axis quantifies the ratio between the actual number of BCL2-GFP cDNA transcripts WT or SNP variant, relative to their corresponding cDNA counting under no treatment (“control”). **h** Relative reference and variant BCL2-GFP transcript concentrations measured by droplet digital PCR. **i** HEK293T cells, grown on coverslips and transfected with reference BCL2-GFP, rs1801018 BCL2-GFP (+ 21 T- > C) or GFP-empty vector. Localization variances between the two BCL2 versions were not observed, protein location is not a factor in the effect of rs1801018. *P*-values were calculated using two-sided *t* test

To validate these findings in clinical settings, we collected formalin-fixed paraffin-embedded (FFPE) specimens of high-grade serous ovarian tumors from 46 patients. We then correlated BCL2 genotype with immunohistochemical staining for BCL2. Figure 5a, b demonstrates the significant differences in staining patterns between representative tumors: the reference wild-type BCL2 genotype did not display staining in tumor cells (infiltrating lymphocytes stained positively), while patients with a C at location 21 of BCL2 (Fig. 5c) displayed positive cytoplasmic staining in tumor cells, indicating the accumulation of BCL2. Figure 5d presents the number of FFPE specimens examined in each group.

### BCL2 with C at location 21 is overexpressed in peripheral blood lymphocytes from ovarian cancer patients

These results suggest a general BCL2-related mechanism. That is, the findings we described so far are not necessarily intrinsic to any cancer mechanism and should be observable in other cell types. Variation at location 21 of BCL2 may regulate the transcriptional activity of the gene in other cell types. To examine this possibility, we collected lymphocytes from the peripheral blood of 12 ovarian cancer patients (seven samples with the reference sequence and five samples with the heterozygous 21 T > C variant), extracted RNA and quantified BCL2 mRNA levels, and compared them to actin using qRT-PCR. BCL2 mRNA levels in lymphocytes from patients with a C instead of a T showed an ~sevenfold increase (*p*-value < 0.01) in BCL2 transcript levels compared with those in samples from reference BCL2 patients (Fig. 5e).

### Cells with a C at location 21 are more resistant to paclitaxel in vitro

These results thus show that cells with a BCL2 sequence with a C at location 21 present a more stable BCL2 transcript, which may lead to higher protein levels. To directly quantify the effect of the variant on the in vitro sensitivity to paclitaxel, the GFP-empty vector control or one of three GFP-BCL2 variants (reference, variant, or random) were overexpressed, and the cells were treated with paclitaxel. Cells were incubated with increasing concentrations of paclitaxel (Fig. 5f). The GFP-empty vector (representing the intrinsic response of HeyA8 cells to paclitaxel) response showed, as expected, high sensitivity to paclitaxel. Cells transfected with either reference or random variation BCL2 showed reduced cell death (increased rates of cell viability), which is also expected, as the levels of BCL2 were elevated compared with the intrinsic response. However, cell viability was significantly improved (*p*-value < 0.05) in cells that were transfected to provide a BCL2 transcript with a 21 T > C.

Another approach to control the expression of the BCL2 transcript is through an increase in copy number. To study the relationship between resistance to paclitaxel and BCL2 gene copy numbers, we performed a BCL2 gradient assay. In such an assay, cell sensitivity to paclitaxel is examined in the presence of increasing levels of BCL2 gene copies. HeyA8 cells were transfected with five different doses of reference BCL2-GFP. Twenty-four hours post transfection, cells were treated with paclitaxel, and 24 h post incubation, we measured cell viability using FACS. As demonstrated in Fig. 5g, an increase in BCL2 copy number led to a decrease in relative cell death in response to paclitaxel.

### Transcripts that include the 21 T > C variant are more abundant in the presence of paclitaxel

To observe transcript differences at the level of the single transcript, we used digital PCR,^39^ which brings potential advantages over real-time PCR, including the ability to obtain absolute quantification without external references and with robustness to variation in PCR efficiency^40^ and an improved dynamic range. We cotransfected cells with the reference and variant forms of BCL2 and treated them with paclitaxel and/or carboplatin. Digital PCR provided a quantified number of mRNA transcripts. The results (Fig. 5h) show that the ratio between transcripts with a 21 T > C and other reference transcripts is preserved, and that the variant form is more abundant. Most importantly, the variant transcript is significantly more abundant when cells are treated with paclitaxel. Under paclitaxel, the reference transcript is practically absent, while the 21 T > C transcript persists. These findings further support our notion that the variant form of the gene leads to higher BCL2 expression, which leads to paclitaxel resistance.

Since differences in localization sometimes produce differences that might lead to observed paclitaxel resistance, we studied BCL2 spatial expression patterns in the reference form and in the 21 T > C form. HEK293T cells were grown on coverslips in a 24-well plate and transfected with the different forms of BCL2, followed by an immunofluorescence assay. Staining of both reference and variant BCL2-GFP demonstrated similar cell localization patterns, whereas the empty vector was localized perfectly in the cell nucleus, thereby ruling out relocalization as a relevant mechanism.

The Broad Institute established a resource to identify drug-targetable dependencies that specific genomic alterations impart on human cancers. CTD2 contains measurements of the sensitivity of hundreds of genetically characterized cancer cell lines to hundreds of small-molecule probes and drugs that have highly selective interactions with their targets.

To test our results in a high-throughput manner, we utilized the CTD2 database,^41,42^ where we examined the area under the curve (AUC) of 39 different ovarian cancer cell lines and 406 different compounds (targeted and cytotoxic) to learn about any associations between the rs1801018 variant and response to different treatments. The z-score was calculated on the AUC levels for every compound. Cell lines with z-scores < −1.5 were tagged as sensitive, and cell lines with z-scores > 0 were tagged as not sensitive.

**Fig. 6:**
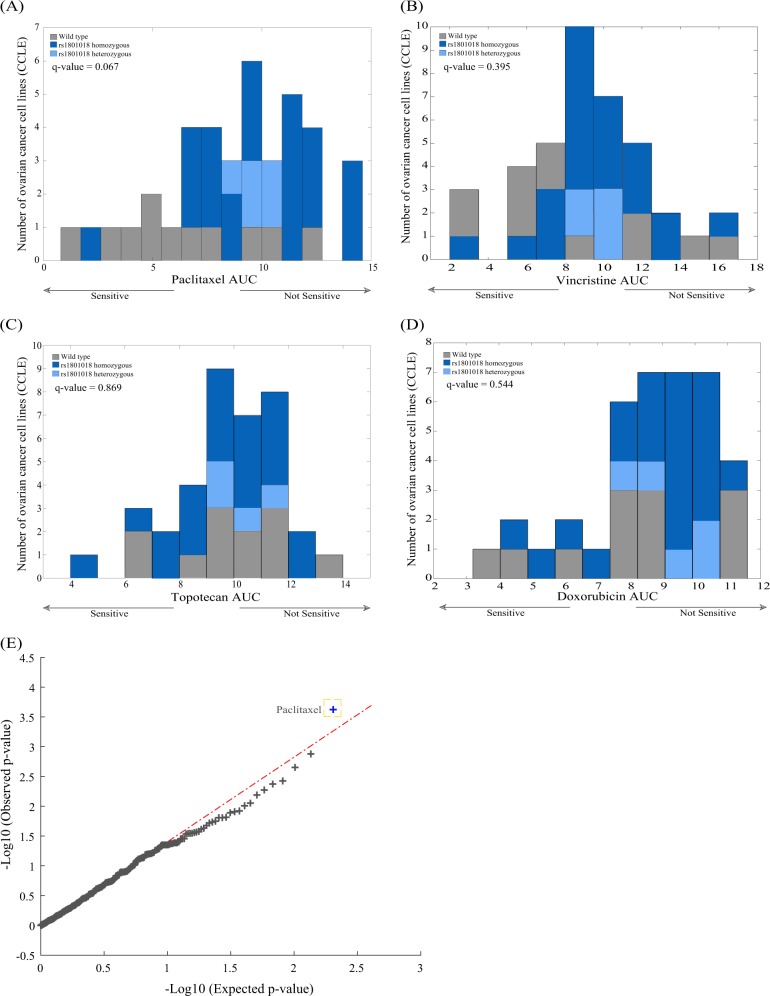
BCL2 variant predict response to paclitaxel in 39 ovarian cancer cell lines. **a** Histogram showing the response of 39 different ovarian cancer cell lines to paclitaxel with their BCL2 variant status (cell lines with wild-type BCL2 marked in gray, heterozygous BCL2 marked in light blue and homozygous for the variant marked in dark blue). Cell lines with low AUC (sensitive to paclitaxel) are enriched with wild-type BCL2, while cells with high AUC (resistant) are enriched with the variant form. **b**–**d** This phenomenon is to observed in other cytotoxic treatment such as vincristine, topotecan, and doxorubicin. **e** This was tested across 406 different treatments, and the only response paclitaxel was the only significant association with the presence of the variant

A chi-square test was performed, and all *p*-values were corrected for multiple hypotheses using the Benjamini–Hochberg procedure. Figure 6a shows the distribution of different BCL2 states (wild-type, heterozygous, and variant) across paclitaxel AUC levels. Low paclitaxel AUC levels (sensitivity) are enriched with cell lines with wild-type BCL2, while high paclitaxel AUC levels (resistance) are enriched with the variant form (q-value = 0.067). Panels b–d in the figure show three additional cytotoxic treatments with no significant association between the status of the variant and the response.

Figure 6e shows the observed *p*-value distribution overlaid over random expected *p*-values. This qq-plot shows that of the 406 different treatments tested, paclitaxel was the only one with a significant value in association with the variant. These results strengthen our findings and show specificity to the paclitaxel response.

## Discussion

For decades, chemotherapy has been performed without the ability to predict the response to platinum/taxane. A personalized treatment in which the patient’s genomic sequence could be used to assign proper therapy may reduce toxicity and costs of treatment and eventually provide an effective treatment.

In the work presented here, we analyzed TCGA data to identify predictive biomarkers for chemotherapy resistance. The results of this analysis show that a single variant in the BCL2 gene is significantly associated with resistance to paclitaxel in multiple tumor types.

We found that the secondary structures of BCL2 variants were dramatically different in conformation. This was supported experimentally by measurements of BCL2 mRNA levels (Fig. 3b) and in analyses of BCL2 mRNA stability following the inhibition of transcription by actinomycin D (Fig. 4a). IN addition, droplet digital PCR results showed higher levels of variant transcripts postchemotherapy treatment compared with reference transcripts. Finally, to observe the effect on protein levels, we showed that the rs1801018 variant produces increased BCL2 protein levels.

To reassociate these molecular findings with clinical findings, we produced an unrelated patient dataset, in which we quantified BCL2 protein levels in FFPE specimens of high-grade serous ovarian tumors. In this additional set of tumors, we showed that while positive staining for BCL2 correlated with the sequence variant genotype, no staining was observed in blocks from patients with the reference genotype (Fig. 5). Furthermore, the quantification of BCL2 mRNA levels in lymphocytes obtained from ovarian cancer patients from Sheba Medical Center displayed a sevenfold increase in expression levels in lymphocytes from patients who presented the BCL2 sequence with the 21 T > C form.

Previous work in this field has shown that a germline deletion in BIM (BCL2L11), which alters RNA splicing and impairs the generation of the death-inducing isoform of BIM, is associated with resistance to epidermal growth factor receptor (EGFR) tyrosine kinase inhibitors (TKIs), such as gefitinib and erlotinib, in lung cancer. It has been reported that patients carrying the BIM deletion allele were more likely to experience acquired gefitinib-resistant disease.^43–46^

Synonymous mutations, which do not alter the protein sequence, have been shown to affect protein function (PMID: 21878961, 24954581, 23901115). Furthermore, Kimchi-Sarfaty et al.^27^ (PMID: 17185560) found that a synonymous SNP in the MDR1 gene alters protein conformation. They hypothesized that the presence of a rare codon, marked by the synonymous polymorphism, affects the timing of cotranslational folding, thereby altering the structure. It is possible that Taxol resistance in the presence of the rs1801018 SNP is not only due to changes in RNA but also to changes in protein conformation.

Precision or personalized medicine is a new emerging area and therapeutic strategy to bring unexpected successes and a new path to improve the treatment and prognosis of patients. The principle of precision medicine is to treat patients based on genetic alterations^47^ after gene mutations are identified. Mutation-based therapy can help better match the drug to the patient. The work presented here associates resistance to paclitaxel with a specific SNP in BCL2. By identifying patients harboring this mutation, we will be able to distinguish the nonresponders and develop better therapies tailored for them.

These data call for a reconsideration of ovarian cancer treatment. Perhaps by considering the patient genotype profile, we may be able to better treat the patient. Above all, these findings invite a search for alternative treatments that can better target the mutated gene. A careful reevaluation of the gold standard treatment is called for. The personalization of treatment and the identification of new drugs that target mutant Bcl2 are needed.

## Materials and methods

### The Cancer Genome Atlas

Data were obtained from The Cancer Genome Atlas (TCGA) database. This dataset comprises of whole-exome sequencing, RNA sequencing and clinical information for 370 ovarian cancer patients, 83 uterine corpus endometrial carcinoma (UCEC) patients, and 28 head and neck squamous cell carcinoma (HNSC), 5 bladder urothelial carcinoma (BLCA), 11 cervical squamous cell carcinoma and endocervical adenocarcinoma (CESC), 4 esophageal carcinoma,^48^ 21 lung adenocarcinoma (LUAD), 13 lung squamous cell carcinoma (LUSC), 2 skin cutaneous melanoma (SKCM), 2 stomach adenocarcinoma (STAD), and 28 uterine carcinosarcoma (UCS) patients. All patients in this paper receives the same first-line treatment composed of the combination of paclitaxel and carboplatin or cisplatin,

### Cell lines and tissue samples

HeyA8 ovarian cell lines (obtained as a generous gift from Prof. Shay Israeli) were grown at 37 °C with 5% CO_2_ in the MEM-EAGLE medium supplement with 2 mM L-glutamine, 1.5 g/L sodium bicarbonate, 4.5 g/L glucose, 10 mM HEPES, 1.0 mM sodium pyruvate, and 10% fetal bovine serum (FBS).

### qRT-PCR

The total RNA extracted from cell lines or lymphocytes were prepared with Trizol reagent according to the manufacturer’s protocol. RNA was subjected to Syber FAST ABI Prism qPCR Kit (KapaBiosystems Inc. Woburn, MA, USA). Reactions were run on 7900HT Real-Time PCR.

Relative expression levels of GFP variants were measured using the following primers:

FF: 5′-TCTGTCTCCGGTGAAGGTGAAG-3′

Rev: 5′-GGCATGGCAGACTTGAAAAAG-3′

Relative expression levels of BCL2 were measured using the following primers:

FF: 5′-AGGCTGGGATGCCTTTGT-3′

Rev: 5′-GACTTCACTTGTGGCCCAGATA-3′

Expression levels were normalized to the actin endogenous control.

### Construction of GFP-BCL2 variants

GFP-BCL2 variants were obtained by introducing point mutations at the following locations: + 21 T > C, + 23 C > T using appropriate primers set and the QuickChange II Site-Directed Mutagenesis Kit (Agilent Technologies) on the GFP-BCL2 variant template, purchased from Addgene (plasmid cat# 3336).

### Western blot

HeyA8 cells were seeded into 10 -cm plates and transfected with GFP-empty vector, GFP-BCL2 reference, variant or a random. Forty-eight hours following transfection, cells were washed with PBS and harvested in M2 lysis buffer (100 mM NaCl, 50 mM Tris, pH 7.5, 1% Triton X-100, 2 mM EDTA) containing protease inhibitor cocktail (Sigma). Extracts were clarified by centrifugation at 12,000×*g* for 15 min at 4 °C, subjected to SDS-PAGE gel and proteins transferred to the nitrocellulose membrane.

The membrane was blocked with 5% low-fat milk and incubated with 1:250 mouse anti-BCL2 (SC-7382) and 1:1000 rabbit anti-GFP- (SC-8334) specific primary antibodies, washed with PBS containing 0.001% Tween20 (PBST), and incubated with the appropriate horseradish peroxidase-conjugated secondary antibodies, mouse HRP (sc-2005), and rabbit HRP (sc-2004), respectively, diluted at 1:10,000. After washing in PBST, the membranes were subjected to enhanced chemiluminescence (ECL) detection analysis.

### mRNA stability

Heya8 cells transfected with GFP or one of three GFP-BCL2 variants (reference, + 21 T > C or random + 23 C > T) were treated 24 h post transfection with Actinomycin D (1 µg/ml, sigma) for 30 min at 37 °C. Cells were treated at the following time points: 0, 2 h, 4 h, and 7 h post incubation period. The total RNA were extracted using TriZol reagent.

### Histological examination

FFPE blocks of high-grade serous ovarian tumors were collected under the approval of the Institutional ethical committee from the archive of the Department of Pathology at the Medical Center. The tumors were removed from patients undergoing either primary cytoreductive or interval debulking surgery at the Medical Center since 2011. To construct a tissue microarray,^49^ representative cores of tissue (1.5 mm in diameter) were taken from each case with a total of 46 different cases. Tumor fragments were rinsed with ice cold PBS. Specimens were fixed in 10% formalin overnight at room temperature and paraffin embedded. Sections of 5 µm were cut using a microtome, deparaffinized and rehydrated through a graded ethanol series, unmasked (Vector H-3300), blocked 2 h in PBS/0.2% Tween20/0.2% gelatin (Sigma G-1890), and stained with hematoxylin and eosin (H&E) for histology examination.

### Immunohistochemistry

For immunohistochemistry analysis, paraffin-embedded 5-µm-thick sections were cut using a microtome. Sections were deparaffinized, heat-induced epitope retrieved and rehydrated through a graded ethanol series, unmasked (Vector H-3300), and blocked 2 h in PBS/0.2% Tween20/0.2% gelatin (Sigma G-1890). Sections were then incubated overnight at 4 °C with monoclonal mouse Anti-Human BCL2 Oncoprotein Clone 124^50^ (1:20) and for an additional 1 h with secondary antibody mouse HRP at room temperature.

### DNA extraction from formaldehyde-fixed, paraffin-embedded (FFPE)

DNA was extracted from FFPEs blocks using DNeasy Blood & Tissue Kit (Qiagen). The recovered DNA has been used as a template for a PCR reaction mediated by KAPA HiFi HotStart ReadyMix PCR Kit (KAPA Biosystem), with the presence of the following BCL2 primer set:

FF: 5′-AGATCTATGGCGCACGCTGGGAGAAC-3′

Rev: 5′-GAATTGTCACTTGTGGCCCAGATAGGC-3′

The yield PCR product was recovered by wizard PCR clean up kit (Promega) and sent for sequence analysis, using the reverse BCL2 primer.

### Cell viability

Propidium iodide^24^ was added to the cell suspension at a concentration of 1 μg/ml. Sorting and analyses were carried out in a Gallios flow cytometer cell sorter collecting 10,000 events. Dead cells were excluded by gating on forward and side scatter and by eliminating PI-positive events.

### BCL2 variant response to Paclitaxel

GFP-empty vector or one of three types of GFP-BCL2 variants were measured for their sensitivity to Paclitaxel. Twenty-four hours post transfections with each of the indicated plasmids, cells were introduced to 100 or 200 nM of Paclitaxel for 24 h incubation.

### In silico RNA analysis

The RNA structure of the reference, variant, and random BCL2 was predicted using the RNA-folding prediction tool Mfold^33,51^ at http://www.bioinfo.rpi.edu/applications/mfold. The significance of changes were quantified to present *p*-values using RNAsnp.^36^

### Droplet digital PCR workflow

Heya8 cells were transfected with reference BCL2-GFP, variant BCL2-GFP. Twenty-four hours post transfection, cells were treated with 30 µM paclitaxel, 232 µM carboplatin, or a combination of them both (IC50 concentration) for additional 24 h. The total RNA were extracted, and the corresponding cDNA was analyzed by droplet digital PCR. The primer mixture includes set of amplicon primers and two probes which were designed to distinguish between the reference and variant forms of the overexpressed transcript:

GFP-BCL2_P1: 5′-TATCGTACCCCGTTCT-3’, GFP-BCL2_P2: 5′-ATCGTACCCTGTTCTC-3′,

GFP-BCL2_R: 5′-CCTCTGCGACAGCTTATAATGGAT-3′, GFP-BCL2_FF: 5’-GAGCTCAAGCTTCGAATTCTGC-3′.

PCR reaction mixture was loaded into the Bio-Rad QX-100 emulsification device, and droplets were generated following the manufacturer’s instructions. The contents were transferred to a 96-well reaction plate and sealed with a pre-heated Eppendorf 96-well heat sealer for 2 s, as recommended by Bio-Rad. PCR was performed as follows: 10 min at 95 °C, 40 cycles each consisting of a 30 s denaturation at 94 °C followed by a 60 °C extension for 60 s, and a final 10 min at 98 °C. After cycling, droplets were analyzed immediately using the Bio-Rad QX-100 Droplet Reader.

### Immunofluorescence

HEK293T cells were grown on coverslips in a 24-well plate and transfected with reference BCL2-GFP, variant BCL2-GFP (+ 21 T- > C) or GFP-empty vector. Forty-eight hours later, cells were fixed for 20 min in PBS containing 4% paraformaldehyde, washed three times with PBS, and permeabilized at the presence of 0.1% Triton X-100 for 10 min. Follow cells were incubated at room temperature with 4-6′ diamidino-2 phenylindole (DAPI, Sigma) to stain cell nuclei. Cells were visualized using a confocal Microscope.

## Supplementary information

SI

## Data Availability

The datasets generated during and/or analyzed during the current study are available in the TCGA repository, https://cancergenome.nih.gov/ The accession number for the pan-cancer samples used in this manuscript is: DbGaP Study Accession phs000178.v10.p8.The data could also be accesses through the National Cancer Institute’s Genomic Data Commons Data Portal https://gdc.cancer.gov/ using the same Accession data phs000178.

## Supplementary information

**Supplementary Information** accompanies the paper on the *npj Precision Oncology* website (10.1038/s41698-019-0084-3).
