# Supplementary material for: Resistance to paclitaxel is associated with a variant of the gene BCL2 in multiple tumor types
Source: NPJ Precis Oncol. 2019 Apr 23;3:12. doi: 10.1038/s41698-019-0084-3 (PMC6478919; doi:10.1038/s41698-019-0084-3)
Supplement: Supplementary file 1 — SI [file 41698_2019_84_MOESM1_ESM.pdf]

Supplementary Figure 1

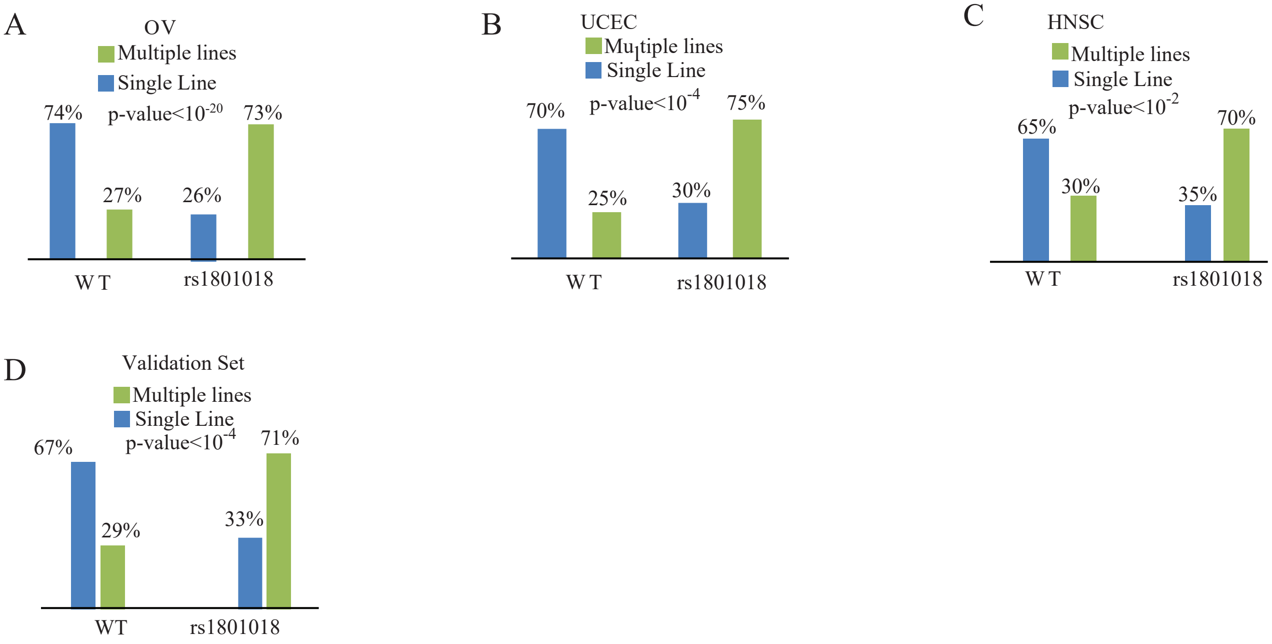

SFigure 1:

The SNP rs1801018 is strongly associated with patient response to paclitaxel treatment. (A) Rs1801018 distribution in response to first line of treatment in ovarian cancer patients. rs1801018 status is highly correlated with the affiliation to the first-line group versus the multiple-line group: out of the 226 patients who required additional lines of treatment, 73 percent (165 patients), displayed T in location 5735 of BCL2; 74 percent (107 patients) of the patients who required a single line of treatment displayed C in this location. In the same manner (B), of 83 UCEC patients, 70 percent of first-line responders displayed the wild-type sequence, while 75 percent (15 out of 20) of multiple-lines patients displayed rs1801018. Panel (C) shows the stratification of the variant as consistent in HNSC (D) and in the validation set used.

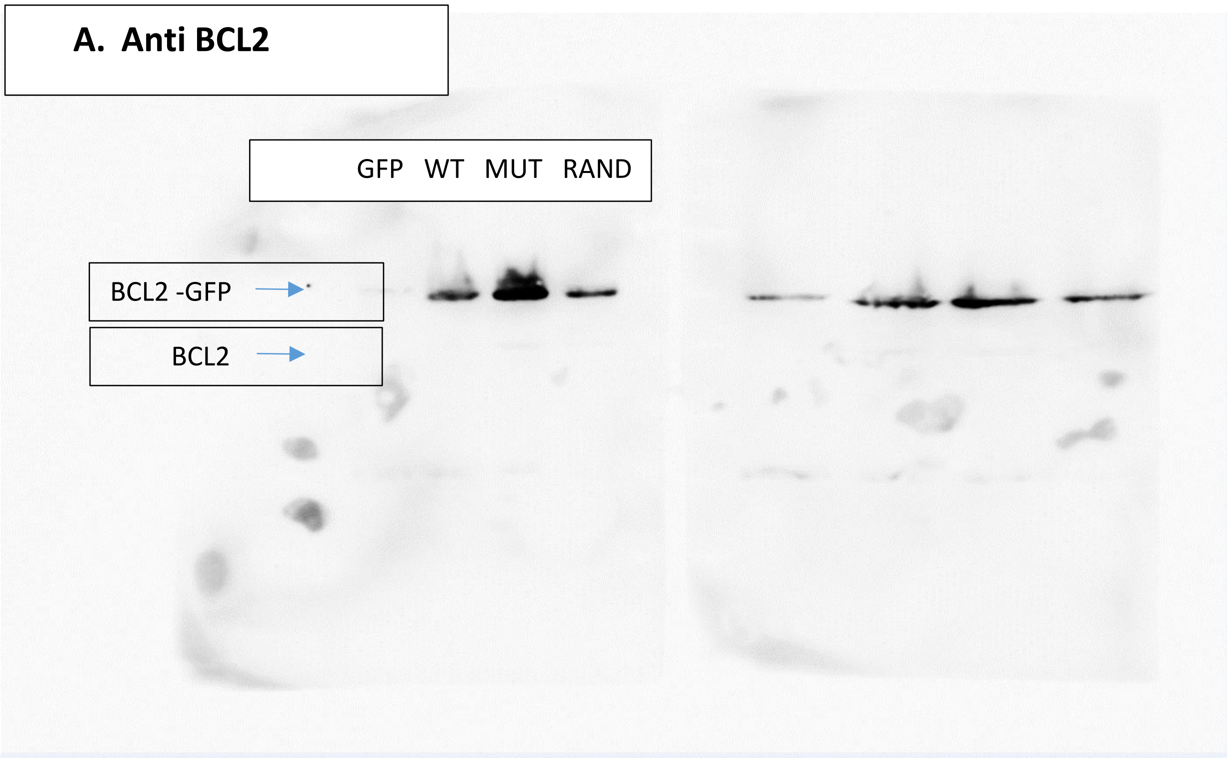
A. Anti BCL2
GFP WT MUT RAND
BCL2 -GFP
BCL2

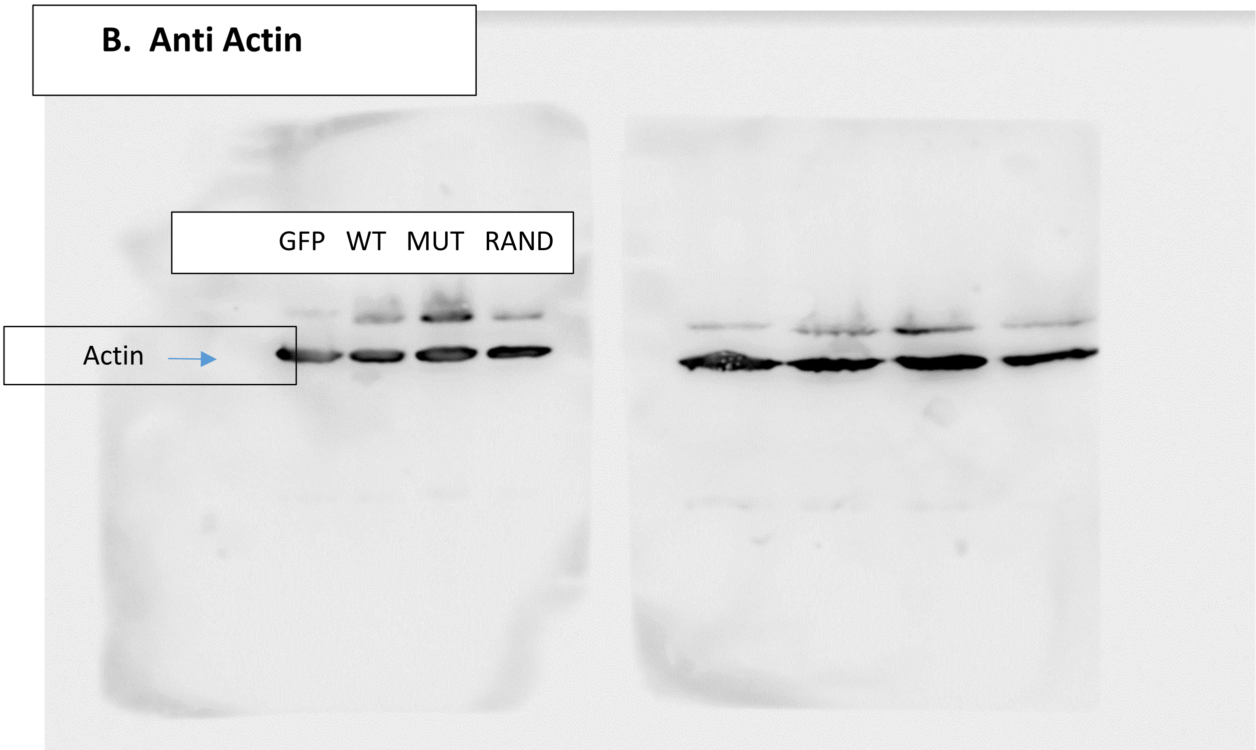
B. Anti Actin
GFP WT MUT RAND
Actin

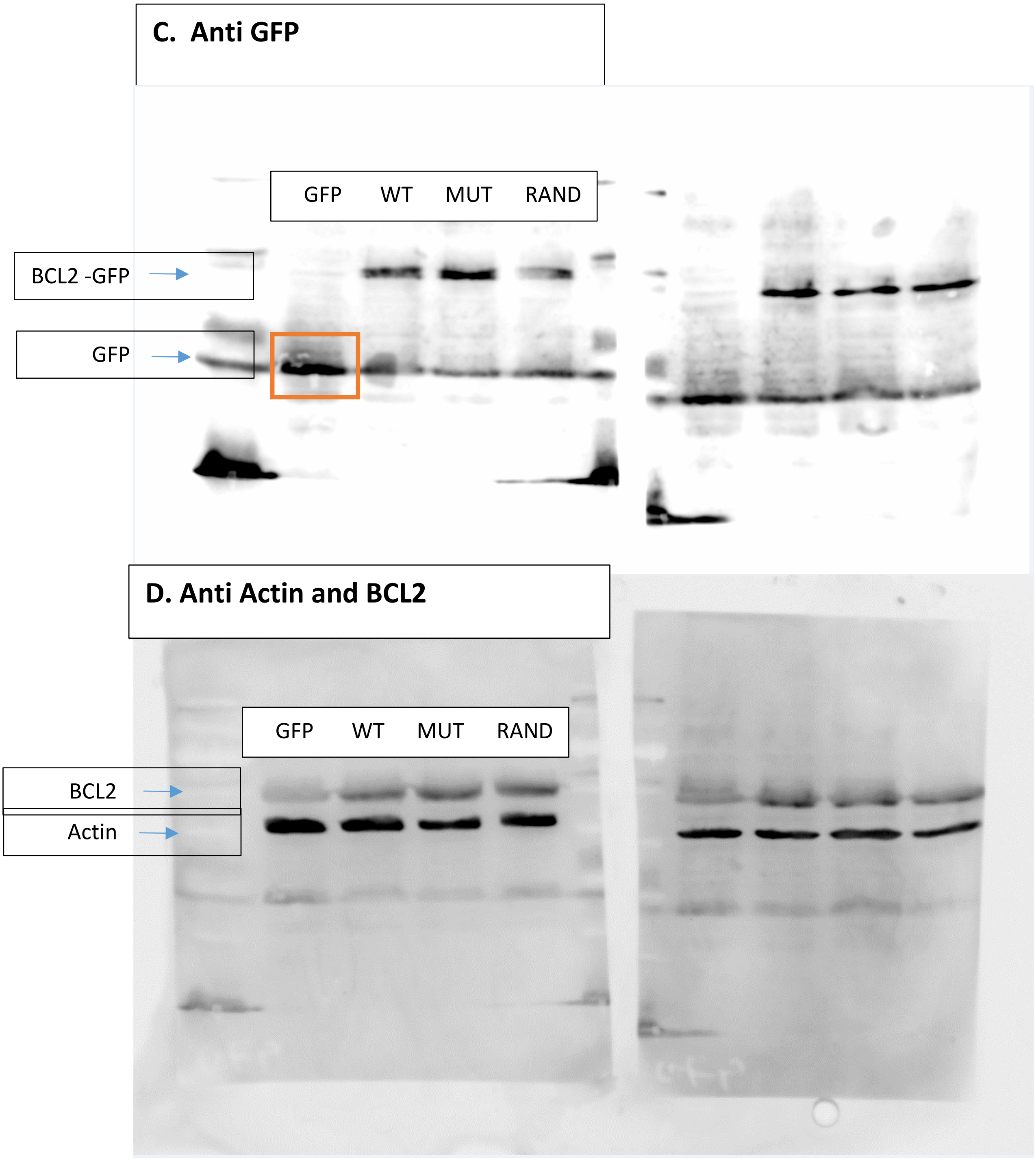

Sup Figure 2 . HEK293T cells were transfected with empty GFP vector (GFP), WT PARP1-GFP (WT), SNP PARP1-GFP (MUT) or with the Random PARP1-GFP (RAND) plasmids. 48h later, lysates were harvested and subjected to SDS-PAGE gel. Proteins were transferred to a nitrocellulose membrane, which was blocked with 3% Milk. **Anti-BCL2** antibody detected the over expressed levels of GFP-BCL2 variants (Left blot) or the endogenous BCL2 (Right blot). Actin measurement served as a loading control for both membranes (B). **Anti-GFP** antibody detected the over expressed levels of GFP-BCL2 variants (Left blot) or empty GFP vector (left and right blots, repeats) (C). The endogenous BCL2 was detected using Anti-BCL2 antibody while anti Actin was measured the endogenous Actin, as a loading control for both membranes (Left and Right) (D).

BCL2 SNPs in ovarian cancer patients from TCGA

Patient ID	Group	rs1801018	rs61733416	rs1800477
Patient 1	single	-	-	-
Patient 10	multiple	+	-	-
Patient 100	single	+	-	-
Patient 101	multiple	+	-	-
Patient 102	single	-	-	-
Patient 103	multiple	+	-	-
Patient 104	multiple	+	-	-
Patient 105	multiple	+	-	-
Patient 106	single	+	-	-
Patient 107	single	+	-	-
Patient 108	single	+	-	-
Patient 109	single	-	-	-
Patient 11	multiple	+	-	-
Patient 110	single	-	-	-
Patient 111	single	-	-	-
Patient 112	single	+	-	-
Patient 113	single	-	-	-
Patient 114	single	-	-	-
Patient 115	single	+	-	-
Patient 116	multiple	+	-	-
Patient 117	single	-	-	-
Patient 118	multiple	+	-	-
Patient 119	multiple	+	-	-
Patient 12	multiple	+	-	-
Patient 120	single	-	-	-
Patient 121	multiple	+	-	-
Patient 122	single	+	-	-
Patient 123	multiple	+	-	-
Patient 124	multiple	-	-	-
Patient 125	multiple	-	-	+
Patient 126	single	-	-	-
Patient 127	single	-	-	-
Patient 128	multiple	+	-	-
Patient 129	single	+	-	-
Patient 13	multiple	+	-	-
Patient 130	multiple	+	-	-
Patient 131	multiple	-	-	-
Patient 132	multiple	+	-	-
Patient 133	multiple	+	-	-
Patient 134	single	-	-	-
Patient 135	single	-	-	-
Patient 136	single	+	-	-
Patient 137	single	+	-	-
Patient 138	single	-	-	-
Patient 139	single	-	-	-
Patient 14	single	-	-	-
Patient 140	multiple	+	-	-
Patient 141	multiple	-	-	-
Patient 142	multiple	+	+	-
Patient 143	single	+	-	-
Patient 144	single	-	-	-

Patient 145	single	+	-	-
Patient 146	single	+	-	-
Patient 147	single	-	-	-
Patient 148	single	-	-	-
Patient 149	multiple	+	-	-
Patient 15	single	-	-	-
Patient 150	multiple	+	-	-
Patient 151	multiple	+	-	-
Patient 152	multiple	+	-	-
Patient 153	multiple	+	-	-
Patient 154	multiple	+	-	-
Patient 155	multiple	+	-	-
Patient 156	single	+	-	-
Patient 157	multiple	+	-	-
Patient 158	multiple	-	-	-
Patient 159	multiple	+	-	-
Patient 16	multiple	+	-	-
Patient 160	multiple	+	-	-
Patient 161	multiple	+	-	-
Patient 162	multiple	+	-	-
Patient 163	multiple	+	-	-
Patient 164	single	-	-	-
Patient 165	single	-	-	-
Patient 166	multiple	+	-	-
Patient 167	multiple	+	-	-
Patient 168	single	-	-	-
Patient 169	multiple	-	-	-
Patient 17	multiple	+	-	-
Patient 170	multiple	+	-	-
Patient 171	single	-	-	-
Patient 172	multiple	+	-	-
Patient 173	multiple	+	-	-
Patient 174	single	-	-	-
Patient 175	multiple	+	-	-
Patient 176	single	-	-	-
Patient 177	multiple	-	-	-
Patient 178	single	-	-	-
Patient 179	multiple	+	-	-
Patient 18	multiple	+	-	-
Patient 180	multiple	+	-	-
Patient 181	multiple	+	-	-
Patient 182	multiple	-	-	-
Patient 183	multiple	-	-	-
Patient 184	single	-	-	-
Patient 185	single	+	-	-
Patient 186	single	-	-	-
Patient 187	single	-	-	-
Patient 188	single	-	-	-
Patient 189	single	+	-	-
Patient 19	multiple	-	-	-
Patient 190	single	-	-	-
Patient 191	single	-	-	-
Patient 192	single	+	-	-

Patient 193	multiple	+	-	-
Patient 194	multiple	+	-	-
Patient 195	multiple	-	-	-
Patient 196	multiple	+	-	-
Patient 197	multiple	+	-	-
Patient 198	multiple	-	-	-
Patient 199	multiple	+	-	-
Patient 2	multiple	+	-	-
Patient 20	multiple	+	-	-
Patient 200	multiple	+	-	-
Patient 201	single	-	-	-
Patient 202	single	+	-	-
Patient 203	multiple	+	-	-
Patient 204	multiple	+	-	-
Patient 205	multiple	+	-	-
Patient 206	single	-	-	-
Patient 207	multiple	+	-	-
Patient 208	multiple	+	-	-
Patient 209	multiple	-	-	-
Patient 21	multiple	+	-	-
Patient 210	multiple	+	-	-
Patient 211	multiple	+	-	-
Patient 212	multiple	+	-	-
Patient 213	multiple	+	-	-
Patient 214	multiple	+	-	-
Patient 215	multiple	+	-	-
Patient 216	multiple	-	-	-
Patient 217	multiple	+	-	-
Patient 218	multiple	+	-	-
Patient 219	multiple	+	-	-
Patient 22	multiple	-	-	-
Patient 220	multiple	-	-	-
Patient 221	multiple	-	-	-
Patient 222	multiple	-	-	-
Patient 223	multiple	+	-	-
Patient 224	single	-	-	-
Patient 225	single	+	-	-
Patient 226	single	+	-	-
Patient 227	single	+	-	-
Patient 228	single	-	-	-
Patient 229	multiple	+	-	-
Patient 23	multiple	+	-	-
Patient 230	multiple	-	-	-
Patient 231	multiple	+	-	-
Patient 232	multiple	-	-	-
Patient 233	single	-	-	-
Patient 234	single	+	-	-
Patient 235	multiple	+	-	-
Patient 236	multiple	+	-	-
Patient 237	multiple	-	+	-
Patient 238	multiple	+	-	-
Patient 239	multiple	+	-	-
Patient 24	single	-	-	-

Patient 240	multiple	+	-	-
Patient 241	multiple	-	-	-
Patient 242	multiple	+	-	-
Patient 243	single	+	-	-
Patient 244	multiple	+	-	-
Patient 245	single	-	-	-
Patient 246	multiple	+	-	-
Patient 247	multiple	-	-	-
Patient 248	multiple	+	-	-
Patient 249	single	-	-	-
Patient 25	single	-	-	-
Patient 250	multiple	+	-	-
Patient 251	multiple	+	-	-
Patient 252	multiple	+	-	-
Patient 253	multiple	+	-	-
Patient 254	multiple	+	-	-
Patient 255	single	-	-	-
Patient 256	multiple	+	-	-
Patient 257	multiple	+	-	-
Patient 258	multiple	+	-	-
Patient 259	multiple	+	-	-
Patient 26	single	-	-	-
Patient 260	multiple	-	-	-
Patient 261	single	-	-	-
Patient 262	multiple	+	-	-
Patient 263	multiple	+	-	-
Patient 264	multiple	+	-	-
Patient 265	multiple	-	+	-
Patient 266	multiple	+	-	-
Patient 267	multiple	+	-	-
Patient 268	multiple	+	-	-
Patient 269	multiple	+	-	-
Patient 27	multiple	+	-	-
Patient 270	multiple	+	-	-
Patient 271	multiple	+	-	-
Patient 272	multiple	-	-	-
Patient 273	multiple	-	+	-
Patient 274	multiple	-	-	-
Patient 275	multiple	+	-	-
Patient 276	multiple	+	-	-
Patient 277	single	-	-	-
Patient 278	single	+	+	-
Patient 279	multiple	+	-	-
Patient 28	single	-	-	-
Patient 280	multiple	-	-	-
Patient 281	multiple	+	-	-
Patient 282	multiple	-	-	-
Patient 283	multiple	-	-	-
Patient 284	single	-	-	-
Patient 285	multiple	-	-	-
Patient 286	multiple	-	-	-
Patient 287	multiple	+	-	-
Patient 288	single	+	-	-

Patient 289	single	-	-	-
Patient 29	multiple	+	-	-
Patient 290	multiple	-	-	-
Patient 291	multiple	+	+	-
Patient 292	single	-	-	-
Patient 293	multiple	+	-	-
Patient 294	multiple	+	-	-
Patient 295	multiple	+	-	-
Patient 296	multiple	-	-	-
Patient 297	single	+	-	-
Patient 298	multiple	+	-	-
Patient 299	single	-	-	-
Patient 3	multiple	-	-	-
Patient 30	multiple	+	-	-
Patient 300	multiple	+	-	-
Patient 301	multiple	+	-	-
Patient 302	multiple	+	-	-
Patient 303	single	-	-	-
Patient 304	single	-	-	-
Patient 305	single	-	-	-
Patient 306	multiple	+	-	-
Patient 307	single	-	-	-
Patient 308	single	-	-	-
Patient 309	single	-	-	-
Patient 31	multiple	+	-	-
Patient 310	multiple	+	-	-
Patient 311	single	-	-	-
Patient 312	multiple	+	-	-
Patient 313	single	+	-	-
Patient 314	single	-	-	-
Patient 315	single	+	-	-
Patient 316	multiple	+	-	-
Patient 317	multiple	+	-	-
Patient 318	multiple	+	-	-
Patient 319	single	-	-	-
Patient 32	multiple	+	-	-
Patient 320	multiple	-	-	-
Patient 321	multiple	+	-	-
Patient 322	multiple	+	-	-
Patient 323	single	-	-	-
Patient 324	single	-	-	-
Patient 325	multiple	-	-	-
Patient 326	single	-	-	-
Patient 327	multiple	-	-	-
Patient 328	single	-	-	-
Patient 329	multiple	-	-	-
Patient 33	multiple	+	-	-
Patient 330	single	-	-	-
Patient 331	single	+	-	-
Patient 332	multiple	+	-	-
Patient 333	multiple	+	-	-
Patient 334	multiple	+	-	-
Patient 335	single	-	-	-

Patient 336	multiple	+	-	-
Patient 337	single	-	-	-
Patient 338	single	+	-	-
Patient 339	single	+	-	-
Patient 34	multiple	+	-	-
Patient 340	multiple	-	-	-
Patient 341	multiple	-	-	-
Patient 342	single	-	-	-
Patient 343	multiple	+	-	-
Patient 344	single	-	-	-
Patient 345	multiple	+	-	-
Patient 346	multiple	+	-	-
Patient 347	multiple	-	-	-
Patient 348	multiple	+	-	-
Patient 349	multiple	-	-	-
Patient 35	single	-	-	-
Patient 350	single	-	-	-
Patient 351	single	-	-	-
Patient 352	single	-	-	-
Patient 353	single	-	-	-
Patient 354	single	-	-	-
Patient 355	single	-	-	-
Patient 356	single	-	-	-
Patient 357	single	-	-	-
Patient 358	multiple	-	-	-
Patient 359	multiple	-	-	-
Patient 36	single	-	-	-
Patient 360	multiple	-	-	-
Patient 361	multiple	-	-	-
Patient 362	multiple	-	-	-
Patient 363	multiple	-	-	-
Patient 364	multiple	-	-	-
Patient 365	multiple	-	-	-
Patient 366	multiple	-	-	-
Patient 367	multiple	+	-	-
Patient 368	multiple	+	-	-
Patient 369	multiple	+	-	-
Patient 37	single	-	-	-
Patient 370	multiple	+	-	-
Patient 38	single	-	-	-
Patient 39	single	-	-	-
Patient 4	multiple	+	-	-
Patient 40	single	-	+	-
Patient 41	multiple	-	-	-
Patient 42	multiple	+	-	-
Patient 43	multiple	+	-	-
Patient 44	multiple	+	-	-
Patient 45	single	-	-	-
Patient 46	single	-	-	-
Patient 47	single	-	-	-
Patient 48	multiple	+	-	-
Patient 49	multiple	-	-	-
Patient 5	multiple	+	-	-

Patient 50	multiple	-	-	-
Patient 51	multiple	-	-	-
Patient 52	single	-	-	-
Patient 53	single	+	-	-
Patient 54	multiple	+	-	-
Patient 55	multiple	-	-	-
Patient 56	single	+	-	-
Patient 57	single	-	-	-
Patient 58	multiple	-	-	-
Patient 59	multiple	+	-	-
Patient 6	single	-	-	-
Patient 60	multiple	+	-	-
Patient 61	single	-	-	-
Patient 62	multiple	+	-	-
Patient 63	multiple	+	-	-
Patient 64	single	-	-	-
Patient 65	single	-	-	-
Patient 66	single	-	-	-
Patient 67	multiple	+	-	-
Patient 68	multiple	+	-	-
Patient 69	multiple	+	-	-
Patient 7	multiple	+	-	-
Patient 70	multiple	+	-	-
Patient 71	single	-	-	-
Patient 72	single	-	-	-
Patient 73	multiple	+	-	-
Patient 74	single	-	-	-
Patient 75	single	-	-	-
Patient 76	single	-	-	-
Patient 77	single	+	-	-
Patient 78	single	+	-	-
Patient 79	single	-	-	-
Patient 8	multiple	+	-	-
Patient 80	multiple	+	-	-
Patient 81	single	-	-	-
Patient 82	single	-	-	-
Patient 83	multiple	-	-	-
Patient 84	multiple	+	-	-
Patient 85	single	-	-	-
Patient 86	multiple	+	-	-
Patient 87	multiple	+	-	-
Patient 88	single	+	-	-
Patient 89	single	-	-	-
Patient 9	multiple	+	-	-
Patient 90	single	-	-	-
Patient 91	multiple	-	-	-
Patient 92	multiple	+	-	-
Patient 93	multiple	+	-	-
Patient 94	single	+	-	-
Patient 95	single	-	-	-
Patient 96	single	-	-	-
Patient 97	single	-	-	-
Patient 98	multiple	+	-	-

Patient 99	multiple	+	-	-

TUBB1 SNPs in ovarian cancer patients from TCGA

Patient ID	Group	rs463312	rs415064	rs35989782	rs34793043	rs41303899	rs163777	rs35565630	rs6070697
Patient 1	single	-	-	-	-	-	+	-	-
Patient 10	multiple	+	+	-	-	-	+	-	-
Patient 100	single	-	-	-	-	-	+	-	-
Patient 101	multiple	-	-	-	-	-	+	-	-
Patient 102	single	-	-	-	-	-	+	-	+
Patient 103	multiple	+	+	-	-	-	+	-	-
Patient 104	multiple	-	-	-	-	-	+	-	-
Patient 105	multiple	-	-	-	-	-	+	-	-
Patient 106	single	-	-	-	-	-	+	-	-
Patient 107	single	-	-	-	-	-	+	-	-
Patient 108	single	-	-	-	-	-	+	-	-
Patient 109	single	-	-	-	-	-	+	-	-
Patient 11	multiple	-	-	-	-	-	+	-	-
Patient 110	single	+	+	-	-	-	+	-	-
Patient 111	single	-	-	-	-	-	+	-	-
Patient 112	single	-	-	-	-	-	+	-	-
Patient 113	single	-	-	-	-	-	+	-	-
Patient 114	single	-	-	-	-	-	+	-	-
Patient 115	single	-	-	-	-	-	+	-	-
Patient 116	multiple	-	-	-	-	-	+	+	-
Patient 117	single	-	-	-	-	-	+	-	-
Patient 118	multiple	+	-	-	-	-	+	-	-
Patient 119	multiple	-	-	-	-	-	+	-	-
Patient 12	multiple	-	-	-	-	-	+	-	-
Patient 120	single	-	-	-	-	-	+	-	-
Patient 121	multiple	-	-	-	-	-	+	-	-
Patient 122	single	-	-	-	-	-	+	-	-
Patient 123	multiple	-	-	-	-	-	+	-	-
Patient 124	multiple	-	-	-	-	-	+	-	-
Patient 125	multiple	-	-	-	-	-	+	-	+
Patient 126	single	-	-	-	-	-	+	-	-
Patient 127	single	+	+	-	-	-	+	-	+
Patient 128	multiple	-	-	-	-	-	+	-	-
Patient 129	single	-	-	-	-	-	+	-	-
Patient 13	multiple	-	-	-	-	-	+	-	-
Patient 130	multiple	-	-	-	-	-	+	-	-
Patient 131	multiple	-	-	-	-	-	+	-	+
Patient 132	multiple	-	-	-	-	-	+	-	+
Patient 133	multiple	-	-	-	-	-	+	-	-
Patient 134	single	-	-	-	-	-	+	-	-
Patient 135	single	-	-	-	-	-	+	-	-
Patient 136	single	-	-	-	-	-	+	-	+
Patient 137	single	-	-	-	-	-	+	-	-
Patient 138	single	-	-	-	-	-	+	-	+
Patient 139	single	-	-	-	-	+	+	-	+
Patient 14	single	-	-	-	-	-	+	-	-
Patient 140	multiple	-	-	-	-	-	+	-	-
Patient 141	multiple	-	-	-	-	-	+	-	-
Patient 142	multiple	-	-	-	-	-	+	-	-
Patient 143	single	-	-	-	-	-	+	-	-
Patient 144	single	-	-	-	-	-	+	-	-

Patient 145	single	-	-	-	+	-	+	-	+
Patient 146	single	-	-	-	-	-	+	-	+
Patient 147	single	-	-	-	-	-	+	-	-
Patient 148	single	-	-	-	-	-	+	-	-
Patient 149	multiple	-	-	-	-	-	+	-	-
Patient 15	single	-	-	-	-	-	+	-	-
Patient 150	multiple	-	-	-	-	-	+	-	-
Patient 151	multiple	-	-	-	-	-	+	-	+
Patient 152	multiple	-	-	-	-	-	+	-	-
Patient 153	multiple	-	-	-	-	-	+	-	-
Patient 154	multiple	-	-	-	-	-	+	-	-
Patient 155	multiple	-	-	-	-	-	+	-	+
Patient 156	single	-	-	-	-	-	+	-	-
Patient 157	multiple	-	-	-	-	-	+	-	-
Patient 158	multiple	-	-	-	-	-	+	-	-
Patient 159	multiple	-	-	-	-	-	+	-	-
Patient 16	multiple	-	-	-	-	-	+	-	-
Patient 160	multiple	-	-	-	-	-	+	-	-
Patient 161	multiple	-	-	-	-	-	+	-	-
Patient 162	multiple	-	-	-	-	-	+	-	-
Patient 163	multiple	-	-	-	-	-	+	-	-
Patient 164	single	-	-	-	-	-	+	-	-
Patient 165	single	-	-	-	-	-	+	-	-
Patient 166	multiple	-	-	-	-	-	+	-	-
Patient 167	multiple	-	-	-	-	-	+	-	-
Patient 168	single	-	-	-	-	-	+	-	+
Patient 169	multiple	-	-	-	-	-	+	-	+
Patient 17	multiple	-	-	-	-	-	+	-	-
Patient 170	multiple	-	-	-	-	-	+	-	-
Patient 171	single	-	-	-	-	-	+	-	+
Patient 172	multiple	-	-	-	-	-	+	-	-
Patient 173	multiple	-	-	-	-	-	+	-	-
Patient 174	single	-	-	-	-	-	+	-	+
Patient 175	multiple	-	-	-	-	-	+	-	-
Patient 176	single	-	-	+	-	-	+	-	+
Patient 177	multiple	-	-	-	-	-	+	-	+
Patient 178	single	-	-	-	-	-	+	-	-
Patient 179	multiple	-	-	-	+	-	+	-	+
Patient 18	multiple	-	-	-	-	-	+	-	-
Patient 180	multiple	-	-	-	-	-	+	-	+
Patient 181	multiple	-	-	-	-	-	+	-	+
Patient 182	multiple	+	+	-	-	-	+	-	+
Patient 183	multiple	-	-	-	-	-	+	-	-
Patient 184	single	-	-	-	-	-	+	-	-
Patient 185	single	-	-	-	-	-	+	-	-
Patient 186	single	-	-	-	-	-	+	-	-
Patient 187	single	-	-	-	-	-	+	-	+
Patient 188	single	-	-	-	-	-	+	-	-
Patient 189	single	-	-	-	-	-	+	-	-
Patient 19	multiple	-	-	-	-	-	+	-	-
Patient 190	single	-	-	-	-	-	+	-	-
Patient 191	single	-	-	-	-	-	+	-	+
Patient 192	single	-	-	-	-	-	+	-	-

Patient 193	multiple	-	-	-	-	-	+	-	-
Patient 194	multiple	-	-	-	-	-	+	-	-
Patient 195	multiple	-	-	-	-	-	+	-	-
Patient 196	multiple	-	-	-	-	-	+	-	+
Patient 197	multiple	-	-	-	-	-	+	-	-
Patient 198	multiple	-	-	-	-	-	+	-	-
Patient 199	multiple	-	-	-	-	-	+	-	+
Patient 2	multiple	-	-	+	-	-	+	-	-
Patient 20	multiple	-	-	-	-	-	+	-	-
Patient 200	multiple	-	-	-	-	-	+	-	-
Patient 201	single	-	-	-	-	-	+	-	-
Patient 202	single	-	-	-	-	-	+	-	+
Patient 203	multiple	-	-	-	-	-	+	-	-
Patient 204	multiple	-	-	-	-	-	+	-	-
Patient 205	multiple	-	-	-	-	-	+	-	-
Patient 206	single	-	-	-	-	-	+	-	-
Patient 207	multiple	-	-	-	-	-	+	-	-
Patient 208	multiple	-	-	-	-	-	+	-	-
Patient 209	multiple	-	-	-	-	-	+	-	-
Patient 21	multiple	-	-	-	-	-	+	-	+
Patient 210	multiple	-	-	-	-	-	+	-	+
Patient 211	multiple	-	-	-	-	-	+	-	-
Patient 212	multiple	-	-	-	-	-	+	-	-
Patient 213	multiple	-	-	-	-	-	+	-	-
Patient 214	multiple	-	-	-	-	-	+	-	-
Patient 215	multiple	-	-	-	-	-	+	-	+
Patient 216	multiple	-	-	+	-	-	+	-	-
Patient 217	multiple	-	-	-	-	-	+	-	-
Patient 218	multiple	-	-	-	-	-	+	-	+
Patient 219	multiple	-	-	-	-	-	+	-	-
Patient 22	multiple	-	-	-	-	-	+	-	+
Patient 220	multiple	-	-	-	-	-	+	-	+
Patient 221	multiple	-	-	-	-	-	+	-	-
Patient 222	multiple	-	-	-	-	-	+	-	-
Patient 223	multiple	-	-	-	-	-	+	-	-
Patient 224	single	+	+	-	-	-	+	-	-
Patient 225	single	-	-	-	-	-	+	-	-
Patient 226	single	+	+	-	-	-	+	-	-
Patient 227	single	-	-	-	-	-	+	-	-
Patient 228	single	-	-	-	-	-	+	-	-
Patient 229	multiple	-	-	-	-	-	+	-	-
Patient 23	multiple	-	-	-	-	-	+	-	-
Patient 230	multiple	-	-	-	-	-	+	-	-
Patient 231	multiple	-	-	-	-	-	+	-	-
Patient 232	multiple	-	-	-	-	-	+	-	-
Patient 233	single	-	-	-	-	-	+	-	+
Patient 234	single	-	-	-	-	-	+	-	-
Patient 235	multiple	-	-	-	-	-	+	-	+
Patient 236	multiple	-	-	-	-	-	+	-	-
Patient 237	multiple	-	-	-	-	-	+	-	+
Patient 238	multiple	-	-	-	-	-	+	-	-
Patient 239	multiple	-	-	-	-	-	+	-	-
Patient 24	single	-	-	-	-	-	+	-	-

Patient 240	multiple	-	-	-	-	-	+	-	-
Patient 241	multiple	-	-	-	-	-	+	-	-
Patient 242	multiple	-	-	-	-	-	+	-	-
Patient 243	single	-	-	-	-	-	+	-	-
Patient 244	multiple	-	-	-	-	-	+	-	-
Patient 245	single	-	-	-	-	-	+	-	+
Patient 246	multiple	-	-	-	-	-	+	-	-
Patient 247	multiple	-	-	-	-	-	+	-	+
Patient 248	multiple	-	-	-	-	-	+	-	-
Patient 249	single	-	-	-	-	-	+	-	+
Patient 25	single	-	-	-	-	-	+	-	-
Patient 250	multiple	-	-	-	-	-	+	-	-
Patient 251	multiple	-	-	+	-	-	+	-	-
Patient 252	multiple	-	-	-	-	-	+	-	-
Patient 253	multiple	-	-	-	-	-	+	-	-
Patient 254	multiple	-	-	-	-	-	+	-	-
Patient 255	single	-	-	-	-	-	+	-	-
Patient 256	multiple	-	-	-	-	-	+	-	-
Patient 257	multiple	-	-	-	-	-	+	-	-
Patient 258	multiple	-	-	-	-	-	+	-	-
Patient 259	multiple	-	-	-	-	-	+	-	-
Patient 26	single	-	-	-	-	-	+	-	-
Patient 260	multiple	-	-	-	-	-	+	-	+
Patient 261	single	-	-	-	-	-	+	-	-
Patient 262	multiple	-	-	-	-	-	+	-	+
Patient 263	multiple	-	-	-	-	-	+	-	-
Patient 264	multiple	-	-	-	-	-	+	-	+
Patient 265	multiple	-	-	-	-	-	+	-	+
Patient 266	multiple	-	-	-	-	-	+	-	-
Patient 267	multiple	-	-	-	+	-	+	-	+
Patient 268	multiple	-	-	-	-	-	+	-	+
Patient 269	multiple	-	-	-	-	-	+	-	-
Patient 27	multiple	-	-	-	-	-	+	-	-
Patient 270	multiple	-	-	-	-	-	+	-	-
Patient 271	multiple	-	-	-	-	-	+	-	+
Patient 272	multiple	-	-	-	-	-	+	-	+
Patient 273	multiple	-	-	-	-	-	+	-	-
Patient 274	multiple	-	-	-	-	-	+	-	-
Patient 275	multiple	-	-	-	-	-	+	-	-
Patient 276	multiple	-	-	-	-	-	+	-	-
Patient 277	single	-	-	-	-	-	+	-	-
Patient 278	single	-	-	-	-	-	+	-	-
Patient 279	multiple	-	-	-	-	-	+	-	-
Patient 28	single	-	-	-	-	-	+	-	-
Patient 280	multiple	-	-	-	-	-	+	-	-
Patient 281	multiple	-	-	+	-	-	+	-	-
Patient 282	multiple	-	-	-	-	-	+	-	-
Patient 283	multiple	-	-	-	-	-	+	-	-
Patient 284	single	-	-	-	-	-	+	-	-
Patient 285	multiple	-	-	-	-	-	+	-	+
Patient 286	multiple	-	-	-	-	-	+	-	+
Patient 287	multiple	-	-	-	-	-	+	-	-
Patient 288	single	-	-	-	-	-	+	-	-

Patient 289	single	-	-	-	-	-	+	-	-
Patient 29	multiple	-	-	-	-	-	+	-	-
Patient 290	multiple	-	-	-	-	-	+	-	-
Patient 291	multiple	-	-	-	-	-	+	-	-
Patient 292	single	-	-	-	-	-	+	-	-
Patient 293	multiple	-	-	-	-	-	+	-	-
Patient 294	multiple	-	-	-	-	-	+	-	-
Patient 295	multiple	-	-	-	-	-	+	-	-
Patient 296	multiple	-	-	-	-	-	+	-	+
Patient 297	single	-	-	-	-	-	+	-	+
Patient 298	multiple	-	-	-	-	-	+	-	-
Patient 299	single	-	-	-	-	-	+	-	-
Patient 3	multiple	-	-	-	-	-	+	-	-
Patient 30	multiple	-	-	-	-	-	+	-	+
Patient 300	multiple	-	-	-	-	-	+	-	-
Patient 301	multiple	-	-	-	-	-	+	-	-
Patient 302	multiple	-	-	-	-	-	+	-	-
Patient 303	single	-	-	-	-	-	+	-	-
Patient 304	single	-	-	-	-	-	+	-	-
Patient 305	single	-	-	-	-	-	+	-	-
Patient 306	multiple	-	-	-	-	-	+	-	-
Patient 307	single	-	-	-	-	-	+	-	+
Patient 308	single	-	-	-	-	-	+	-	-
Patient 309	single	-	-	-	-	-	+	-	-
Patient 31	multiple	-	-	-	-	-	+	-	+
Patient 310	multiple	-	-	-	-	-	+	-	-
Patient 311	single	-	-	-	-	-	+	-	-
Patient 312	multiple	-	-	-	-	-	+	-	-
Patient 313	single	-	-	-	-	-	+	-	-
Patient 314	single	-	-	-	-	-	+	-	+
Patient 315	single	-	-	-	-	-	+	-	-
Patient 316	multiple	-	-	-	-	-	+	-	+
Patient 317	multiple	+	+	-	-	-	+	-	-
Patient 318	multiple	-	-	-	-	-	+	-	-
Patient 319	single	-	-	-	-	-	+	-	-
Patient 32	multiple	-	-	-	-	-	+	-	+
Patient 320	multiple	-	-	-	-	-	+	-	+
Patient 321	multiple	-	-	-	-	-	+	-	-
Patient 322	multiple	-	-	-	-	-	+	-	-
Patient 323	single	-	-	-	-	-	+	-	+
Patient 324	single	-	-	-	-	-	+	-	+
Patient 325	multiple	-	-	+	-	-	+	-	-
Patient 326	single	-	-	-	-	-	+	-	-
Patient 327	multiple	+	+	-	-	-	+	-	-
Patient 328	single	-	-	-	-	-	+	-	-
Patient 329	multiple	-	-	+	-	-	+	-	-
Patient 33	multiple	-	-	-	-	-	+	-	-
Patient 330	single	-	-	-	-	-	+	-	-
Patient 331	single	-	-	-	-	-	+	-	-
Patient 332	multiple	-	-	-	-	-	+	-	-
Patient 333	multiple	-	-	-	-	-	+	-	-
Patient 334	multiple	-	-	-	-	-	+	-	-
Patient 335	single	-	-	-	-	-	+	-	-

Patient 336	multiple	-	-	-	-	-	+	-	-
Patient 337	single	-	-	-	-	-	+	-	-
Patient 338	single	-	-	-	-	-	+	-	-
Patient 339	single	+	+	-	-	-	+	-	-
Patient 34	multiple	-	-	-	-	-	+	-	-
Patient 340	multiple	-	-	-	-	-	+	-	+
Patient 341	multiple	+	+	-	-	-	+	-	-
Patient 342	single	-	-	-	-	-	+	-	-
Patient 343	multiple	-	-	-	-	-	+	-	+
Patient 344	single	-	-	-	-	-	+	-	+
Patient 345	multiple	-	-	-	-	-	+	-	-
Patient 346	multiple	-	-	-	-	-	+	-	+
Patient 347	multiple	-	-	-	-	-	+	-	-
Patient 348	multiple	-	-	-	-	-	+	-	-
Patient 349	multiple	-	-	-	-	-	+	-	-
Patient 35	single	-	-	-	-	-	+	-	+
Patient 350	single	+	+	-	-	-	+	-	-
Patient 351	single	-	-	-	-	-	+	-	-
Patient 352	single	-	-	-	-	-	+	-	+
Patient 353	single	-	-	-	-	-	+	-	+
Patient 354	single	-	-	-	-	-	+	-	-
Patient 355	single	-	-	-	-	-	+	-	-
Patient 356	single	-	-	-	-	-	+	-	-
Patient 357	single	-	-	-	-	-	+	-	-
Patient 358	multiple	-	-	-	-	-	+	-	-
Patient 359	multiple	-	-	-	-	-	+	-	-
Patient 36	single	-	-	-	-	-	+	-	+
Patient 360	multiple	-	-	-	-	-	+	-	-
Patient 361	multiple	-	-	-	-	-	+	-	-
Patient 362	multiple	-	-	-	-	-	+	-	-
Patient 363	multiple	-	-	-	-	-	+	-	-
Patient 364	multiple	-	-	-	-	-	+	-	-
Patient 365	multiple	-	-	-	-	-	+	-	-
Patient 366	multiple	-	-	-	-	-	+	-	-
Patient 367	multiple	-	-	-	-	-	+	-	+
Patient 368	multiple	-	-	-	-	-	+	-	+
Patient 369	multiple	+	+	-	-	-	+	-	-
Patient 37	single	-	-	-	-	-	+	-	-
Patient 370	multiple	-	-	-	-	-	+	-	-
Patient 38	single	-	-	-	-	-	+	-	-
Patient 39	single	-	-	-	-	-	+	-	+
Patient 4	multiple	-	-	-	-	-	+	-	-
Patient 40	single	+	+	-	-	-	+	-	-
Patient 41	multiple	-	-	-	-	-	+	-	-
Patient 42	multiple	-	-	-	-	-	+	-	+
Patient 43	multiple	-	-	-	-	-	+	-	+
Patient 44	multiple	-	-	-	-	-	+	-	+
Patient 45	single	-	-	-	-	-	+	-	+
Patient 46	single	-	-	-	-	-	+	-	-
Patient 47	single	-	-	-	-	-	+	-	+
Patient 48	multiple	-	-	-	-	-	+	-	-
Patient 49	multiple	-	-	-	-	-	+	-	-
Patient 5	multiple	+	+	-	-	-	+	-	-

Patient 50	multiple	-	-	-	-	-	+	-	+
Patient 51	multiple	-	-	-	-	-	+	-	-
Patient 52	single	-	-	-	-	-	+	-	-
Patient 53	single	-	-	-	-	-	+	-	-
Patient 54	multiple	-	-	-	-	-	+	-	-
Patient 55	multiple	-	-	-	-	-	+	-	-
Patient 56	single	-	-	-	-	-	+	-	-
Patient 57	single	-	-	-	-	-	+	-	+
Patient 58	multiple	-	-	-	-	-	+	-	-
Patient 59	multiple	-	-	-	-	-	+	-	-
Patient 6	single	-	-	-	-	-	+	-	+
Patient 60	multiple	-	-	-	-	-	+	-	-
Patient 61	single	-	-	-	-	-	+	-	-
Patient 62	multiple	-	-	-	-	-	+	-	+
Patient 63	multiple	-	-	-	-	-	+	-	+
Patient 64	single	-	-	-	-	-	+	-	-
Patient 65	single	-	-	-	-	-	+	-	-
Patient 66	single	-	-	+	-	-	+	-	-
Patient 67	multiple	-	-	-	-	-	+	-	-
Patient 68	multiple	-	-	-	-	-	+	-	-
Patient 69	multiple	-	-	-	-	-	+	-	-
Patient 7	multiple	-	-	-	-	-	+	-	-
Patient 70	multiple	-	-	-	-	-	+	-	+
Patient 71	single	-	-	-	-	-	+	-	-
Patient 72	single	-	-	-	-	-	+	-	-
Patient 73	multiple	-	-	-	-	-	+	-	-
Patient 74	single	-	-	-	-	-	+	-	-
Patient 75	single	-	-	-	-	-	+	-	-
Patient 76	single	-	-	-	-	-	+	-	-
Patient 77	single	-	-	-	-	-	+	-	+
Patient 78	single	-	-	-	-	-	+	-	-
Patient 79	single	-	-	-	-	-	+	-	-
Patient 8	multiple	-	-	-	-	-	+	-	+
Patient 80	multiple	-	-	-	-	-	+	-	+
Patient 81	single	-	-	-	-	-	+	-	-
Patient 82	single	-	-	-	-	-	+	-	-
Patient 83	multiple	-	-	-	-	-	+	-	-
Patient 84	multiple	-	-	-	-	-	+	-	+
Patient 85	single	-	-	-	-	-	+	-	-
Patient 86	multiple	-	-	-	-	-	+	-	+
Patient 87	multiple	-	-	-	+	-	+	-	-
Patient 88	single	-	-	-	-	-	+	-	+
Patient 89	single	-	-	-	-	-	+	-	+
Patient 9	multiple	-	-	-	-	-	+	-	-
Patient 90	single	-	-	-	-	-	+	-	+
Patient 91	multiple	-	-	-	-	-	+	-	-
Patient 92	multiple	-	-	-	-	-	+	-	-
Patient 93	multiple	-	-	-	-	-	+	-	+
Patient 94	single	-	-	-	-	-	+	-	-
Patient 95	single	-	-	-	-	-	+	-	-
Patient 96	single	-	-	-	-	-	+	-	-
Patient 97	single	-	-	-	-	-	+	-	-
Patient 98	multiple	-	-	-	-	-	+	-	-

Patient 99	multiple	-	-	-	-	-	+	-	-

BCL2 SNPs in cancer patients from TCGA

Cancer Type	Patient ID	Group	rs1801018	rs61733416	rs1800477
BLCA	Patient 1	Single	-	-	-
BLCA	Patient 2	Single	+	-	-
BLCA	Patient 3	Single	+	-	-
BLCA	Patient 4	Single	+	-	-
CESC	Patient 5	Single	-	-	-
CESC	Patient 6	Single	-	-	-
CESC	Patient 7	Single	-	-	-
CESC	Patient 8	Single	-	-	-
CESC	Patient 9	Single	-	-	-
CESC	Patient 10	Single	-	-	-
CESC	Patient 11	Single	-	-	-
CESC	Patient 12	Single	-	-	-
ESCA	Patient 13	Single	-	+	-
ESCA	Patient 14	Single	-	-	-
ESCA	Patient 15	Single	-	-	-
HNSC	Patient 16	Single	-	-	-
HNSC	Patient 17	Single	-	-	-
HNSC	Patient 18	Single	-	-	-
HNSC	Patient 19	Single	-	-	-
HNSC	Patient 20	Single	-	-	-
HNSC	Patient 21	Single	-	-	-
HNSC	Patient 22	Single	-	-	-
HNSC	Patient 23	Single	-	-	-
HNSC	Patient 24	Single	-	-	-
HNSC	Patient 25	Single	-	-	-
HNSC	Patient 26	Single	-	-	-
HNSC	Patient 27	Single	+	-	-
HNSC	Patient 28	Single	+	-	-
HNSC	Patient 29	Single	+	-	-
HNSC	Patient 30	Single	+	-	-
HNSC	Patient 31	Single	+	-	-
HNSC	Patient 32	Single	+	-	-
LUAD	Patient 33	Single	-	-	-
LUAD	Patient 34	Single	-	-	-
LUAD	Patient 35	Single	-	-	-
LUAD	Patient 36	Single	-	-	-
LUAD	Patient 37	Single	-	-	-
LUAD	Patient 38	Single	+	+	-
LUAD	Patient 39	Single	+	+	-
LUAD	Patient 40	Single	+	-	-
LUAD	Patient 41	Single	+	-	-
LUAD	Patient 42	Single	+	-	-
LUSC	Patient 43	Single	-	-	-
LUSC	Patient 44	Single	-	-	-
LUSC	Patient 45	Single	-	-	-
LUSC	Patient 46	Single	-	-	-
LUSC	Patient 47	Single	-	-	-
LUSC	Patient 48	Single	-	-	-
LUSC	Patient 49	Single	+	-	-
LUSC	Patient 50	Single	+	-	-
SKCM	Patient 51	Single	+	-	-

STAD	Patient 52	Single	-	-	-
STAD	Patient 53	Single	-	-	+
UCEC	Patient 54	Single	-	-	-
UCEC	Patient 55	Single	-	-	-
UCEC	Patient 56	Single	-	-	-
UCEC	Patient 57	Single	-	-	-
UCEC	Patient 58	Single	-	-	-
UCEC	Patient 59	Single	-	-	-
UCEC	Patient 60	Single	-	-	-
UCEC	Patient 61	Single	-	-	+
UCEC	Patient 62	Single	-	-	-
UCEC	Patient 63	Single	-	-	-
UCEC	Patient 64	Single	-	+	-
UCEC	Patient 65	Single	-	-	-
UCEC	Patient 66	Single	-	-	-
UCEC	Patient 67	Single	-	+	-
UCEC	Patient 68	Single	-	-	-
UCEC	Patient 69	Single	-	-	-
UCEC	Patient 70	Single	-	-	-
UCEC	Patient 71	Single	-	-	-
UCEC	Patient 72	Single	-	-	-
UCEC	Patient 73	Single	-	-	-
UCEC	Patient 74	Single	-	-	-
UCEC	Patient 75	Single	-	-	-
UCEC	Patient 76	Single	-	-	-
UCEC	Patient 77	Single	-	-	-
UCEC	Patient 78	Single	-	-	-
UCEC	Patient 79	Single	-	-	-
UCEC	Patient 80	Single	-	-	-
UCEC	Patient 81	Single	-	-	-
UCEC	Patient 82	Single	-	-	-
UCEC	Patient 83	Single	-	-	-
UCEC	Patient 84	Single	-	-	-
UCEC	Patient 85	Single	-	-	-
UCEC	Patient 86	Single	-	-	-
UCEC	Patient 87	Single	-	-	-
UCEC	Patient 88	Single	-	-	-
UCEC	Patient 89	Single	-	-	-
UCEC	Patient 90	Single	-	-	-
UCEC	Patient 91	Single	-	-	-
UCEC	Patient 92	Single	-	-	-
UCEC	Patient 93	Single	-	-	-
UCEC	Patient 94	Single	-	-	-
UCEC	Patient 95	Single	-	-	-
UCEC	Patient 96	Single	-	-	-
UCEC	Patient 97	Single	-	-	-
UCEC	Patient 98	Single	+	-	-
UCEC	Patient 99	Single	+	-	-
UCEC	Patient 100	Single	+	-	-
UCEC	Patient 101	Single	+	-	-
UCEC	Patient 102	Single	+	-	-
UCEC	Patient 103	Single	+	-	-
UCEC	Patient 104	Single	+	-	-

UCEC	Patient 105	Single	+	-	-
UCEC	Patient 106	Single	+	-	-
UCEC	Patient 107	Single	+	-	-
UCEC	Patient 108	Single	+	-	-
UCEC	Patient 109	Single	+	-	-
UCEC	Patient 110	Single	+	-	-
UCEC	Patient 111	Single	+	-	-
UCEC	Patient 112	Single	+	-	-
UCEC	Patient 113	Single	+	-	-
UCEC	Patient 114	Single	+	-	-
UCEC	Patient 115	Single	+	-	-
UCEC	Patient 116	Single	+	-	-
UCS	Patient 117	Single	-	-	-
UCS	Patient 118	Single	-	-	-
UCS	Patient 119	Single	-	-	-
UCS	Patient 120	Single	-	-	-
UCS	Patient 121	Single	-	-	-
UCS	Patient 122	Single	-	-	-
UCS	Patient 123	Single	-	-	-
UCS	Patient 124	Single	-	-	-
UCS	Patient 125	Single	-	-	-
UCS	Patient 126	Single	-	-	-
UCS	Patient 127	Single	-	-	-
UCS	Patient 128	Single	-	-	-
UCS	Patient 129	Single	+	-	-
UCS	Patient 130	Single	+	-	-
UCS	Patient 131	Single	+	-	-
UCS	Patient 132	Single	+	-	-
UCS	Patient 133	Single	+	-	-
UCS	Patient 134	Single	+	-	-
UCS	Patient 135	Single	+	-	-
BLCA	Patient 136	Multiple	+	-	-
CESC	Patient 137	Multiple	-	-	-
CESC	Patient 138	Multiple	-	-	-
ESCA	Patient 139	Multiple	+	-	-
HNSC	Patient 140	Multiple	-	-	-
HNSC	Patient 141	Multiple	-	-	-
HNSC	Patient 142	Multiple	-	-	-
HNSC	Patient 143	Multiple	+	-	-
HNSC	Patient 144	Multiple	+	-	-
HNSC	Patient 145	Multiple	+	-	-
LUAD	Patient 146	Multiple	-	-	-
LUAD	Patient 147	Multiple	-	-	-
LUAD	Patient 148	Multiple	-	-	-
LUAD	Patient 149	Multiple	+	-	-
LUAD	Patient 150	Multiple	+	-	-
LUAD	Patient 151	Multiple	+	-	-
LUAD	Patient 152	Multiple	+	-	-
LUAD	Patient 153	Multiple	+	-	-
LUSC	Patient 154	Multiple	-	-	-
LUSC	Patient 155	Multiple	-	-	-
LUSC	Patient 156	Multiple	+	-	-
SKCM	Patient 157	Multiple	+	-	-

UCEC	Patient 158	Multiple	-	-	-
UCEC	Patient 159	Multiple	-	-	-
UCEC	Patient 160	Multiple	+	-	-
UCEC	Patient 161	Multiple	+	-	-
UCEC	Patient 162	Multiple	+	-	-
UCEC	Patient 163	Multiple	+	-	-
UCEC	Patient 164	Multiple	+	-	-
UCEC	Patient 165	Multiple	+	-	-
UCEC	Patient 166	Multiple	+	-	-
UCEC	Patient 167	Multiple	+	-	-
UCEC	Patient 168	Multiple	+	-	-
UCEC	Patient 169	Multiple	+	-	-
UCS	Patient 170	Multiple	+	-	-
UCS	Patient 171	Multiple	+	-	-
UCS	Patient 172	Multiple	+	-	-
UCS	Patient 173	Multiple	+	-	-
UCS	Patient 174	Multiple	+	-	-
UCS	Patient 175	Multiple	+	-	-
HNSC	Patient 176	Multiple	+	-	-
HNSC	Patient 177	Multiple	+	-	-
LUAD	Patient 178	Multiple	+	-	-
LUSC	Patient 179	Multiple	+	-	-
UCEC	Patient 180	Multiple	-	-	-
UCEC	Patient 181	Multiple	+	-	-
UCEC	Patient 182	Multiple	+	-	-
UCEC	Patient 183	Multiple	+	-	-
UCS	Patient 184	Multiple	-	-	-
UCS	Patient 185	Multiple	+	-	-
UCS	Patient 186	Multiple	+	-	-
CESC	Patient 187	Multiple	+	-	-
LUAD	Patient 188	Multiple	+	-	-
LUAD	Patient 189	Multiple	+	-	-
LUSC	Patient 190	Multiple	-	-	-
UCEC	Patient 191	Multiple	-	-	-
UCEC	Patient 192	Multiple	+	-	-
UCEC	Patient 193	Multiple	+	-	-
HNSC	Patient 194	Multiple	+	-	-
UCEC	Patient 195	Multiple	-	-	-
HNSC	Patient 196	Multiple	+	+	-

TUBB1 SNPs in cancer patients from TCGA

Cancer	Patient ID	Group	rs463312 + rs415064	rs35989782	rs34793043	rs41303899	rs163777	rs35565630	rs6070697
BLCA	Patient 1	Single	-	-	-	-	+	-	-
BLCA	Patient 2	Single	-	-	-	-	+	-	-
BLCA	Patient 3	Single	-	-	-	-	+	-	-
BLCA	Patient 4	Single	-	-	-	-	+	-	-
CESC	Patient 5	Single	-	-	-	-	+	-	+
CESC	Patient 6	Single	-	-	-	-	+	-	-
CESC	Patient 7	Single	-	-	-	-	+	-	+
CESC	Patient 8	Single	-	-	-	-	+	-	-
CESC	Patient 9	Single	-	-	-	-	+	-	-
CESC	Patient 10	Single	-	-	-	-	-	-	-
CESC	Patient 11	Single	-	-	-	-	-	-	-
CESC	Patient 12	Single	-	-	-	-	-	-	-
ESCA	Patient 13	Single	-	-	-	-	-	-	-
ESCA	Patient 14	Single	-	-	-	-	-	-	-
ESCA	Patient 15	Single	-	-	-	-	-	-	-
HNSC	Patient 16	Single	+	-	-	-	+	-	-
HNSC	Patient 17	Single	+	-	-	-	+	-	-
HNSC	Patient 18	Single	-	-	-	-	+	-	+
HNSC	Patient 19	Single	-	-	-	-	+	-	+
HNSC	Patient 20	Single	-	-	-	-	+	-	-
HNSC	Patient 21	Single	-	-	-	-	+	-	-
HNSC	Patient 22	Single	-	-	-	-	+	-	+
HNSC	Patient 23	Single	+	-	-	-	+	-	+
HNSC	Patient 24	Single	-	-	-	-	+	-	-
HNSC	Patient 25	Single	+	-	-	-	+	-	-
HNSC	Patient 26	Single	+	-	-	-	+	-	+
HNSC	Patient 27	Single	+	-	-	-	+	-	-
HNSC	Patient 28	Single	-	-	-	-	+	-	+
HNSC	Patient 29	Single	-	-	-	-	+	-	-
HNSC	Patient 30	Single	-	-	-	-	+	-	+
HNSC	Patient 31	Single	+	-	-	-	+	-	+
HNSC	Patient 32	Single	-	-	-	-	+	+	+
LUAD	Patient 33	Single	-	-	-	-	+	-	-
LUAD	Patient 34	Single	-	-	-	-	+	-	-
LUAD	Patient 35	Single	-	-	-	-	+	-	+
LUAD	Patient 36	Single	-	-	-	-	+	-	-
LUAD	Patient 37	Single	-	-	-	-	+	-	+
LUAD	Patient 38	Single	-	-	-	-	+	-	-
LUAD	Patient 39	Single	-	-	-	-	+	-	-
LUAD	Patient 40	Single	-	-	-	-	+	-	-
LUAD	Patient 41	Single	-	-	-	-	+	-	-
LUAD	Patient 42	Single	-	-	-	-	+	-	+
LUSC	Patient 43	Single	-	-	-	-	+	-	-
LUSC	Patient 44	Single	-	-	-	-	+	-	+
LUSC	Patient 45	Single	+	-	-	-	+	-	-
LUSC	Patient 46	Single	-	-	-	-	+	-	+
LUSC	Patient 47	Single	+	-	-	-	+	-	-
LUSC	Patient 48	Single	-	-	-	-	+	-	+
LUSC	Patient 49	Single	-	-	-	-	+	-	-
LUSC	Patient 50	Single	-	-	-	-	+	-	+
SKCM	Patient 51	Single	-	-	-	-	+	-	-

STAD	Patient 52	Single	-	-	-	-	+	-	-
STAD	Patient 53	Single	-	-	-	-	+	-	-
UCEC	Patient 54	Single	-	-	-	-	+	-	+
UCEC	Patient 55	Single	-	-	-	-	+	-	+
UCEC	Patient 56	Single	-	-	-	-	+	-	+
UCEC	Patient 57	Single	-	-	-	-	+	-	-
UCEC	Patient 58	Single	-	-	-	-	+	-	-
UCEC	Patient 59	Single	-	-	-	-	+	-	-
UCEC	Patient 60	Single	-	-	-	-	+	-	-
UCEC	Patient 61	Single	-	-	-	-	+	-	-
UCEC	Patient 62	Single	+	-	-	-	+	-	-
UCEC	Patient 63	Single	-	-	+	-	+	-	+
UCEC	Patient 64	Single	-	-	-	-	+	-	-
UCEC	Patient 65	Single	-	-	-	-	+	-	+
UCEC	Patient 66	Single	-	-	-	-	+	-	-
UCEC	Patient 67	Single	-	-	-	-	+	-	+
UCEC	Patient 68	Single	+	-	-	-	+	-	-
UCEC	Patient 69	Single	-	-	-	-	+	-	+
UCEC	Patient 70	Single	-	-	-	-	+	-	-
UCEC	Patient 71	Single	-	-	-	-	+	-	-
UCEC	Patient 72	Single	+	-	-	-	+	-	-
UCEC	Patient 73	Single	-	-	-	-	+	-	-
UCEC	Patient 74	Single	-	-	-	-	+	-	+
UCEC	Patient 75	Single	-	-	-	-	+	-	-
UCEC	Patient 76	Single	+	-	-	-	+	-	+
UCEC	Patient 77	Single	-	-	-	-	+	-	-
UCEC	Patient 78	Single	-	-	-	-	+	-	+
UCEC	Patient 79	Single	-	-	-	-	+	-	-
UCEC	Patient 80	Single	-	-	-	-	+	+	+
UCEC	Patient 81	Single	-	-	-	-	+	-	-
UCEC	Patient 82	Single	+	-	-	-	+	-	-
UCEC	Patient 83	Single	-	-	-	-	+	-	-
UCEC	Patient 84	Single	-	-	-	-	+	-	-
UCEC	Patient 85	Single	-	-	-	-	+	-	-
UCEC	Patient 86	Single	-	-	-	-	+	-	-
UCEC	Patient 87	Single	-	-	-	-	+	-	-
UCEC	Patient 88	Single	-	-	-	-	+	-	-
UCEC	Patient 89	Single	-	-	-	-	+	-	-
UCEC	Patient 90	Single	-	-	-	-	+	-	-
UCEC	Patient 91	Single	+	-	-	-	+	-	-
UCEC	Patient 92	Single	-	-	-	-	+	-	+
UCEC	Patient 93	Single	-	-	-	-	+	-	+
UCEC	Patient 94	Single	-	-	-	-	+	-	-
UCEC	Patient 95	Single	-	-	-	-	+	-	-
UCEC	Patient 96	Single	-	-	-	-	+	-	-
UCEC	Patient 97	Single	-	-	-	-	+	-	-
UCEC	Patient 98	Single	-	-	-	-	+	-	+
UCEC	Patient 99	Single	+	-	-	-	+	-	-
UCEC	Patient 100	Single	-	-	-	-	+	-	+
UCEC	Patient 101	Single	-	-	-	-	+	-	+
UCEC	Patient 102	Single	-	-	-	-	+	-	-
UCEC	Patient 103	Single	-	-	-	-	+	-	-
UCEC	Patient 104	Single	-	-	-	-	+	-	-

UCEC	Patient 105	Single	-	-	-	-	+	-	-
UCEC	Patient 106	Single	-	-	-	-	+	-	-
UCEC	Patient 107	Single	+	-	-	-	+	-	-
UCEC	Patient 108	Single	-	-	-	-	+	-	+
UCEC	Patient 109	Single	-	-	-	-	+	-	+
UCEC	Patient 110	Single	+	-	-	-	+	-	-
UCEC	Patient 111	Single	-	-	-	-	+	-	+
UCEC	Patient 112	Single	+	-	-	-	+	-	-
UCEC	Patient 113	Single	-	-	-	-	+	-	-
UCEC	Patient 114	Single	-	-	-	-	+	-	+
UCEC	Patient 115	Single	-	-	-	-	+	-	-
UCEC	Patient 116	Single	+	-	-	-	+	-	-
UCS	Patient 117	Single	-	-	-	-	+	-	-
UCS	Patient 118	Single	-	-	-	-	+	-	-
UCS	Patient 119	Single	+	-	-	-	+	-	+
UCS	Patient 120	Single	-	-	-	-	+	-	-
UCS	Patient 121	Single	-	-	-	-	+	-	-
UCS	Patient 122	Single	+	-	-	-	+	-	+
UCS	Patient 123	Single	-	-	-	-	+	-	-
UCS	Patient 124	Single	-	-	-	-	+	-	-
UCS	Patient 125	Single	+	-	-	-	+	-	-
UCS	Patient 126	Single	-	-	-	-	+	-	-
UCS	Patient 127	Single	-	-	-	-	+	-	+
UCS	Patient 128	Single	+	-	-	-	+	-	-
UCS	Patient 129	Single	-	-	-	-	+	-	-
UCS	Patient 130	Single	-	-	-	-	+	-	-
UCS	Patient 131	Single	-	-	-	-	+	-	+
UCS	Patient 132	Single	+	-	-	-	+	-	+
UCS	Patient 133	Single	-	-	-	-	+	-	-
UCS	Patient 134	Single	-	-	-	-	-	-	-
UCS	Patient 135	Single	-	-	-	-	+	-	-
BLCA	Patient 136	Multiple	-	-	-	-	+	-	-
CESC	Patient 137	Multiple	-	-	-	-	+	-	-
CESC	Patient 138	Multiple	-	-	-	-	+	-	-
ESCA	Patient 139	Multiple	-	-	-	-	+	-	-
HNSC	Patient 140	Multiple	-	-	-	-	+	-	-
HNSC	Patient 141	Multiple	-	-	-	-	+	-	-
HNSC	Patient 142	Multiple	-	-	-	-	+	-	+
HNSC	Patient 143	Multiple	-	-	-	-	+	-	-
HNSC	Patient 144	Multiple	-	-	-	-	+	-	-
HNSC	Patient 145	Multiple	-	-	-	-	+	-	-
LUAD	Patient 146	Multiple	-	-	-	-	+	-	-
LUAD	Patient 147	Multiple	-	-	-	-	+	-	-
LUAD	Patient 148	Multiple	-	-	-	-	+	-	+
LUAD	Patient 149	Multiple	-	-	-	-	+	-	-
LUAD	Patient 150	Multiple	-	-	-	-	+	-	-
LUAD	Patient 151	Multiple	-	-	-	-	+	-	+
LUAD	Patient 152	Multiple	-	-	-	-	+	-	+
LUAD	Patient 153	Multiple	-	-	-	-	+	-	-
LUSC	Patient 154	Multiple	-	-	-	-	+	-	+
LUSC	Patient 155	Multiple	-	-	-	-	+	-	-
LUSC	Patient 156	Multiple	+	+	-	-	+	-	-
SKCM	Patient 157	Multiple	-	-	-	-	+	-	-

UCEC	Patient 158	Multiple	-	-	-	-	+	-	-
UCEC	Patient 159	Multiple	+	-	-	-	+	-	-
UCEC	Patient 160	Multiple	-	-	-	-	+	-	-
UCEC	Patient 161	Multiple	-	-	-	-	+	-	-
UCEC	Patient 162	Multiple	-	-	-	-	+	-	-
UCEC	Patient 163	Multiple	-	-	-	-	+	+	+
UCEC	Patient 164	Multiple	+	-	-	-	+	-	-
UCEC	Patient 165	Multiple	+	-	-	-	+	-	-
UCEC	Patient 166	Multiple	+	-	-	-	+	-	-
UCEC	Patient 167	Multiple	-	-	-	-	+	-	-
UCEC	Patient 168	Multiple	-	-	-	-	+	-	-
UCEC	Patient 169	Multiple	-	-	-	-	-	-	-
UCS	Patient 170	Multiple	-	-	-	-	-	-	-
UCS	Patient 171	Multiple	-	-	-	-	-	-	-
UCS	Patient 172	Multiple	-	-	-	-	-	-	-
UCS	Patient 173	Multiple	-	-	-	-	-	-	-
UCS	Patient 174	Multiple	-	-	-	-	+	-	-
UCS	Patient 175	Multiple	-	-	-	-	-	-	-
HNSC	Patient 176	Multiple	-	-	-	-	+	-	-
HNSC	Patient 177	Multiple	-	-	-	-	+	-	-
LUAD	Patient 178	Multiple	-	-	-	-	+	-	+
LUSC	Patient 179	Multiple	-	-	-	-	-	-	-
UCEC	Patient 180	Multiple	-	-	-	-	+	-	-
UCEC	Patient 181	Multiple	-	-	-	-	+	-	-
UCEC	Patient 182	Multiple	-	-	-	-	+	-	-
UCEC	Patient 183	Multiple	-	-	-	-	-	-	-
UCS	Patient 184	Multiple	-	-	-	-	+	-	-
UCS	Patient 185	Multiple	-	-	-	-	+	-	+
UCS	Patient 186	Multiple	-	-	-	-	+	-	-
CESC	Patient 187	Multiple	-	-	-	-	+	-	-
LUAD	Patient 188	Multiple	-	-	-	-	+	-	-
LUAD	Patient 189	Multiple	-	-	-	-	+	-	-
LUSC	Patient 190	Multiple	-	-	-	-	-	-	-
UCEC	Patient 191	Multiple	-	-	-	-	-	-	-
UCEC	Patient 192	Multiple	-	-	-	-	-	-	-
UCEC	Patient 193	Multiple	-	-	-	-	+	-	+
HNSC	Patient 194	Multiple	-	-	-	-	+	-	-
UCEC	Patient 195	Multiple	-	-	-	-	-	-	-
HNSC	Patient 196	Multiple	-	-	-	-	-	-	-

SNP ID	Gene Symbol	OV	UCEC	HNSC	Validation Set
rs463312	TUBB1	0.7579	0.6223	0.5891	0.8858
rs415064	TUBB1	0.5859	0.4564	0.711	0.6953
rs35989782	TUBB1	0.4124	0.1741	-	-
rs34793043	TUBB1	0.5645	-	-	0.1803
rs41303899	TUBB1	0.201	-	-	-
rs163777	TUBB1	1	0.5708	1	0.6458
rs35565630	TUBB1	0.4235	0.1741	-	0.1803
rs6070697	TUBB1	0.9617	0.1906	0.2466	0.137
rs1801018	BCL2	1.00E-16	0.0003	0.05	0.0006
rs61733416	BCL2	0.5688	0.4198	0.1839	0.1856
rs1800477	BCL2	0.4235	0.1741	-	0.4501

Patient ID	Ovarian Cancer BCL2 Genotype	BCL2 normalized_count
Patient 1	0/0	35.2046
Patient 2	0/0	40.3493
Patient 3	0/0	44.5173
Patient 4	0/0	46.4037
Patient 5	0/0	52.9291
Patient 6	0/0	54.5682
Patient 7	0/0	57.0356
Patient 8	0/0	70.1589
Patient 9	0/0	74.641
Patient 10	0/0	75.52
Patient 11	0/0	78.758
Patient 12	0/0	82.6798
Patient 13	0/0	82.7912
Patient 14	0/0	88.058
Patient 15	0/0	88.6766
Patient 16	0/0	88.9548
Patient 17	0/0	89.5425
Patient 18	0/0	96.815
Patient 19	0/0	97.5898
Patient 20	0/0	100.8984
Patient 21	0/0	103.9584
Patient 22	0/0	105.6099
Patient 23	0/0	105.7778
Patient 24	0/0	106.6914
Patient 25	0/0	108.464
Patient 26	0/0	109.668
Patient 27	0/0	110.7741
Patient 28	0/0	117.2733
Patient 29	0/0	121.078
Patient 30	0/0	121.2471
Patient 31	0/0	122.9725
Patient 32	0/0	131.9902
Patient 33	0/0	134.9045
Patient 34	0/0	136.954
Patient 35	0/0	144.0114
Patient 36	0/0	155.7487
Patient 37	0/0	156.28
Patient 38	0/0	159.2308
Patient 39	0/0	164.9123
Patient 40	0/0	166.9809
Patient 41	0/0	177.3298
Patient 42	0/0	177.8218
Patient 43	0/0	178.1132
Patient 44	0/0	180.4933
Patient 45	0/0	194.0809
Patient 46	0/0	203.092
Patient 47	0/0	219.9255
Patient 48	0/0	231.3612
Patient 49	0/0	241.7795
Patient 50	0/0	248.5108
Patient 51	0/0	253.125

Patient 52	0/0	260.6534
Patient 53	0/0	265.4919
Patient 54	0/0	266.4395
Patient 55	0/0	268.7525
Patient 56	0/0	284.3489
Patient 57	0/0	284.8012
Patient 58	0/0	289.0533
Patient 59	0/0	291.2072
Patient 60	0/0	310.9436
Patient 61	0/0	329.0503
Patient 62	0/0	344.9643
Patient 63	0/0	356.6094
Patient 64	0/0	360.1023
Patient 65	0/0	375
Patient 66	0/0	401.1434
Patient 67	0/0	404.5888
Patient 68	0/0	420.2195
Patient 69	0/0	428.3831
Patient 70	0/0	444.4271
Patient 71	0/0	462.5889
Patient 72	0/0	486.4061
Patient 73	0/0	508.1055
Patient 74	0/0	558.304
Patient 75	0/0	558.304
Patient 76	0/0	646.6629
Patient 77	1/0	88.0322
Patient 78	1/0	96.8002
Patient 79	1/0	335.3812
Patient 80	1/0	81.4593
Patient 81	1/0	128.6503
Patient 82	1/0	215.4991
Patient 83	1/0	104.4731
Patient 84	1/0	123.1119
Patient 85	1/0	51.715
Patient 86	1/0	298.0296
Patient 87	1/0	216.674
Patient 88	1/0	84.9277
Patient 89	1/0	91.5896
Patient 90	1/0	116.2815
Patient 91	1/0	116.2342
Patient 92	1/0	419.5786
Patient 93	1/0	175.2705
Patient 94	1/0	114.9693
Patient 95	1/0	162.7466
Patient 96	1/0	872.0236
Patient 97	1/0	384.6553
Patient 98	1/0	107.5616
Patient 99	1/0	541.4353
Patient 100	1/0	30.8036
Patient 101	1/0	403.2099
Patient 102	1/0	154.5491
Patient 103	1/0	585.3338
Patient 104	1/0	90.4977

Patient 105	1/0	205.098
Patient 106	1/0	182.0677
Patient 107	1/0	191.1432
Patient 108	1/0	110.3268
Patient 109	1/0	520.3641
Patient 110	1/0	1124.928
Patient 111	1/0	209.2755
Patient 112	1/0	134.9681
Patient 113	1/0	189.9939
Patient 114	1/0	102.7163
Patient 115	1/0	248.1415
Patient 116	1/0	535.3798
Patient 117	1/0	181.0345
Patient 118	1/0	389.4831
Patient 119	1/0	491.4371
Patient 120	1/0	79.974
Patient 121	1/0	164.4568
Patient 122	1/0	153.1972
Patient 123	1/1	211.856
Patient 124	1/1	90.0729
Patient 125	1/1	24.4691
Patient 126	1/1	363.7343
Patient 127	1/1	413.7316
Patient 128	1/1	205.5331
Patient 129	1/1	69.9639
Patient 130	1/1	140.2075
Patient 131	1/1	232.7458
Patient 132	1/1	461.179
Patient 133	1/1	159.3458
Patient 134	1/1	120.724
Patient 135	1/1	140.5579
Patient 136	1/1	205.5116
Patient 137	1/1	1565.6579
Patient 138	1/1	325.9865
Patient 139	1/1	57.9603
Patient 140	1/1	197.4835
Patient 141	1/1	326.6916
Patient 142	1/1	57.6583
Patient 143	1/1	544.6168
Patient 144	1/1	774.4361
Patient 145	1/1	149.0245
Patient 146	1/1	94.8478
Patient 147	1/1	1240.9798
Patient 148	1/1	45.5874
Patient 149	1/1	86.4947
Patient 150	1/1	328.9251
Patient 151	1/1	143.6576
Patient 152	1/1	212.6544
Patient 153	1/1	496.1202
Patient 154	1/1	209.2455
Patient 155	1/1	110.9282
Patient 156	1/1	130.9297

Patient ID	Breast Cancer BCL2 Genotype	BCL2 normalized_count
Patient 1	0/0	822.8869
Patient 2	0/0	1235.0888
Patient 3	0/0	205.2667
Patient 4	0/0	165.4669
Patient 5	0/0	1178.208
Patient 6	0/0	2010.0901
Patient 7	0/0	1459.1918
Patient 8	0/0	165.3384
Patient 9	0/0	484.8157
Patient 10	0/0	1170.126
Patient 11	0/0	234.4525
Patient 12	0/0	1211.126
Patient 13	0/0	2058.7762
Patient 14	0/0	1557.8465
Patient 15	0/0	3491.9095
Patient 16	0/0	161.8404
Patient 17	0/0	185.3377
Patient 18	0/0	1708.299
Patient 19	0/0	491.9608
Patient 20	0/0	1469.1513
Patient 21	0/0	787.6291
Patient 22	0/0	2368.9119
Patient 23	0/0	1735.3879
Patient 24	0/0	1747.4067
Patient 25	0/0	954.7206
Patient 26	0/0	359.9298
Patient 27	0/0	3614.3449
Patient 28	0/0	1580.2831
Patient 29	0/0	2831.4033
Patient 30	0/0	3596.8109
Patient 31	0/0	680.7982
Patient 32	0/0	447.3423
Patient 33	0/0	239.0033
Patient 34	0/0	1197.0173
Patient 35	0/0	125.9267
Patient 36	0/0	1540.1606
Patient 37	0/0	1578.9921
Patient 38	0/0	466.7445
Patient 39	0/0	1982.3398
Patient 40	0/0	1120.5357
Patient 41	0/0	1230.2947
Patient 42	0/0	983.6
Patient 43	0/0	597.8923
Patient 44	0/0	610.7584
Patient 45	0/0	354.3304
Patient 46	0/0	1509.9313
Patient 47	0/0	283.5728
Patient 48	0/0	1260.0508
Patient 49	0/0	2440.2073
Patient 50	0/0	1198.0754
Patient 51	0/0	957.3488

Patient 52	0/0	2850.5747
Patient 53	0/0	732.2714
Patient 54	0/0	281.4861
Patient 55	0/0	3611.5044
Patient 56	0/0	1804.0422
Patient 57	0/0	3258.7367
Patient 58	0/0	3715.0327
Patient 59	0/0	425.5701
Patient 60	0/0	3282.7131
Patient 61	0/0	3021.1222
Patient 62	0/0	269.9952
Patient 63	0/0	992.3445
Patient 64	0/0	72.1038
Patient 65	0/0	1655.2927
Patient 66	0/0	3828.38
Patient 67	0/0	284.4266
Patient 68	0/0	755.4585
Patient 69	0/0	11213.2899
Patient 70	0/0	2184.3425
Patient 71	0/0	67.531
Patient 72	0/0	296.7306
Patient 73	0/0	1973.8632
Patient 74	0/0	1076.0812
Patient 75	0/0	2572.6795
Patient 76	0/0	408.5193
Patient 77	0/0	1954.6273
Patient 78	0/0	333.7832
Patient 79	0/0	1777.7778
Patient 80	0/0	1556.541
Patient 81	0/0	1645.4171
Patient 82	0/0	392.6469
Patient 83	0/0	4303.5557
Patient 84	0/0	682.6404
Patient 85	0/0	316.2791
Patient 86	0/0	1912.7444
Patient 87	0/0	1150.0816
Patient 88	0/0	281.7342
Patient 89	0/0	1225.8315
Patient 90	0/0	381.9943
Patient 91	0/0	2479.9298
Patient 92	0/0	505.6818
Patient 93	0/0	1797.3535
Patient 94	0/0	1215.077
Patient 95	0/0	6005.7504
Patient 96	0/0	342.7946
Patient 97	0/0	1614.415
Patient 98	0/0	1268.8998
Patient 99	0/0	2612.8431
Patient 100	0/0	928.5965
Patient 101	0/0	368.2081
Patient 102	0/0	1613.2906
Patient 103	0/0	466.9876
Patient 104	0/0	3554.1538

Patient 105	0/0	2631.8629
Patient 106	0/0	2891.2368
Patient 107	0/0	987.5071
Patient 108	0/0	516.8904
Patient 109	0/0	1016.9654
Patient 110	0/0	3135.2657
Patient 111	0/0	177.3002
Patient 112	0/0	928.955
Patient 113	0/0	8127.7687
Patient 114	0/0	934.2708
Patient 115	0/0	2085.9873
Patient 116	0/0	2569.3582
Patient 117	0/0	194.422
Patient 118	0/0	472.3655
Patient 119	0/0	1266.7885
Patient 120	0/0	2798.2234
Patient 121	0/0	4535.7143
Patient 122	0/0	3683.0023
Patient 123	0/0	1120.6397
Patient 124	0/0	3164.7165
Patient 125	0/0	208.7708
Patient 126	0/0	2246.5303
Patient 127	0/0	2468.9241
Patient 128	0/0	5070.7806
Patient 129	0/0	330.8958
Patient 130	0/0	2869.7213
Patient 131	0/0	1880.2626
Patient 132	0/0	397.2047
Patient 133	0/0	4309.4213
Patient 134	0/0	2397.7619
Patient 135	0/0	1717.4505
Patient 136	0/0	4060.5204
Patient 137	0/0	1811.7155
Patient 138	0/0	1634.1626
Patient 139	0/0	4147.3725
Patient 140	0/0	2360.4882
Patient 141	0/0	150.5152
Patient 142	0/0	1273.8329
Patient 143	0/0	2844.1298
Patient 144	0/0	1899.5542
Patient 145	0/0	2154.4589
Patient 146	0/0	2244.9105
Patient 147	0/0	140.0871
Patient 148	0/0	2478.6859
Patient 149	0/0	348.7829
Patient 150	0/0	146.3508
Patient 151	0/0	321.218
Patient 152	0/0	957.7991
Patient 153	0/0	2050.173
Patient 154	0/0	502.8054
Patient 155	0/0	1716.929
Patient 156	0/0	87.523
Patient 157	0/0	86.6218

Patient 158	0/0	319.1444
Patient 159	0/0	277.8802
Patient 160	0/0	132.7669
Patient 161	0/0	412.8304
Patient 162	0/0	1046.0663
Patient 163	0/0	732.0599
Patient 164	0/0	625.947
Patient 165	0/0	2483.4773
Patient 166	0/0	3365.4092
Patient 167	0/0	263.6693
Patient 168	0/0	3140.6194
Patient 169	0/0	305.239
Patient 170	0/0	331.1132
Patient 171	0/0	1925.3474
Patient 172	0/0	332.3651
Patient 173	0/0	749.8998
Patient 174	0/0	199.8398
Patient 175	0/0	875.8138
Patient 176	0/0	3897.4605
Patient 177	0/0	990.3264
Patient 178	0/0	154.5791
Patient 179	0/0	1023.8569
Patient 180	0/0	2652.0993
Patient 181	0/0	1379.0573
Patient 182	0/0	3185.3302
Patient 183	0/0	1741.5553
Patient 184	0/1	1860.3509
Patient 185	0/1	2553.836
Patient 186	0/1	3726.9834
Patient 187	0/1	3001.1423
Patient 188	0/1	2363.8354
Patient 189	0/1	4792.265
Patient 190	0/1	1914.276
Patient 191	0/1	370.869
Patient 192	0/1	5037.159
Patient 193	0/1	610.999
Patient 194	0/1	833.4559
Patient 195	0/1	1898.467
Patient 196	0/1	9403.1101
Patient 197	0/1	77.992
Patient 198	0/1	4114.4836
Patient 199	0/1	260.8234
Patient 200	0/1	492.4798
Patient 201	0/1	654.341
Patient 202	0/1	1373.758
Patient 203	0/1	637.5532
Patient 204	0/1	1192.857
Patient 205	0/1	1162.023
Patient 206	0/1	3781.6786
Patient 207	0/1	2704.918
Patient 208	0/1	1622.2966
Patient 209	0/1	3116.4669
Patient 210	0/1	952.3245

Patient 211	0/1	2606.4231
Patient 212	0/1	1196.2751
Patient 213	0/1	998.0797
Patient 214	0/1	4836.4817
Patient 215	0/1	905.1233
Patient 216	0/1	520.6254
Patient 217	0/1	1551.7644
Patient 218	0/1	4149.5816
Patient 219	0/1	2891.2806
Patient 220	0/1	89.9005
Patient 221	0/1	4667.9927
Patient 222	0/1	73.8495
Patient 223	0/1	1788.368
Patient 224	0/1	643.4034
Patient 225	0/1	3655.5204
Patient 226	0/1	474.8161
Patient 227	0/1	1311.2844
Patient 228	0/1	262.9731
Patient 229	0/1	1447.94
Patient 230	0/1	115.3319
Patient 231	0/1	968.0404
Patient 232	0/1	226.1808
Patient 233	0/1	637.11
Patient 234	0/1	1797.4635
Patient 235	0/1	1472.2083
Patient 236	0/1	2589.8499
Patient 237	0/1	832.0023
Patient 238	0/1	1828.8
Patient 239	0/1	1332.0374
Patient 240	0/1	2818.2409
Patient 241	0/1	948.7825
Patient 242	0/1	3800.3423
Patient 243	0/1	372.4364
Patient 244	0/1	1143.1808
Patient 245	0/1	393.4763
Patient 246	0/1	540.6345
Patient 247	0/1	1537.4899
Patient 248	0/1	522.3536
Patient 249	0/1	2161.3807
Patient 250	0/1	2069.2719
Patient 251	0/1	756.4411
Patient 252	0/1	1758.5809
Patient 253	0/1	890.7104
Patient 254	0/1	3626.8452
Patient 255	0/1	1099.1004
Patient 256	0/1	3954.3714
Patient 257	0/1	1922.9442
Patient 258	0/1	2209.5177
Patient 259	0/1	708.1075
Patient 260	0/1	2762.3443
Patient 261	0/1	6792.3461
Patient 262	0/1	3182.4052
Patient 263	0/1	1578.9334

Patient 264	0/1	1681.8562
Patient 265	0/1	5026.1345
Patient 266	0/1	739.8109
Patient 267	0/1	65.9662
Patient 268	0/1	2635.4647
Patient 269	0/1	371.8434
Patient 270	0/1	1109.8784
Patient 271	0/1	777.9451
Patient 272	0/1	2376.696
Patient 273	0/1	210.8283
Patient 274	0/1	634.2629
Patient 275	0/1	3555.6863
Patient 276	0/1	2651.2426
Patient 277	0/1	1684.9421
Patient 278	0/1	3868.7827
Patient 279	0/1	1276.0173
Patient 280	0/1	3178.7837
Patient 281	0/1	159.2955
Patient 282	0/1	220.9979
Patient 283	0/1	3155.8923
Patient 284	0/1	2715.3951
Patient 285	0/1	1612.5615
Patient 286	0/1	2067.5566
Patient 287	0/1	518.9344
Patient 288	0/1	3886.6797
Patient 289	0/1	2527.7457
Patient 290	0/1	739.4451
Patient 291	0/1	662.3409
Patient 292	0/1	2635.3448
Patient 293	0/1	1107.932
Patient 294	0/1	2219.7802
Patient 295	0/1	2308.0737
Patient 296	0/1	118.3746
Patient 297	0/1	3293.7543
Patient 298	0/1	2150.547
Patient 299	0/1	555.0239
Patient 300	0/1	2584.3528
Patient 301	0/1	2248.3131
Patient 302	0/1	1397.1061
Patient 303	0/1	1202.6195
Patient 304	0/1	2909.6735
Patient 305	0/1	2355.7809
Patient 306	0/1	3766.8867
Patient 307	0/1	4500.2647
Patient 308	0/1	1166.9277
Patient 309	0/1	611.508
Patient 310	0/1	1838.731
Patient 311	0/1	582.9894
Patient 312	0/1	723.6842
Patient 313	0/1	3573.8653
Patient 314	0/1	5255.9545
Patient 315	0/1	4571.9587
Patient 316	0/1	640.6626

Patient 317	0/1	236.7323
Patient 318	0/1	903.4874
Patient 319	0/1	880.998
Patient 320	0/1	3097.3312
Patient 321	0/1	2372.0565
Patient 322	0/1	1009.5071
Patient 323	0/1	7208.1641
Patient 324	0/1	1967.8363
Patient 325	0/1	781.0491
Patient 326	0/1	969.8131
Patient 327	0/1	5728.519
Patient 328	0/1	924.5734
Patient 329	0/1	2382.2536
Patient 330	0/1	272.423
Patient 331	0/1	1106.9495
Patient 332	0/1	1597.1816
Patient 333	0/1	9213.9776
Patient 334	0/1	1562.9032
Patient 335	0/1	2054.3807
Patient 336	0/1	1225.4098
Patient 337	0/1	1978.0733
Patient 338	0/1	3155.7178
Patient 339	0/1	1317.4615
Patient 340	0/1	1253.2717
Patient 341	0/1	165
Patient 342	0/1	471.5828
Patient 343	0/1	2376.7969
Patient 344	0/1	493.1643
Patient 345	0/1	6498.4584
Patient 346	0/1	38.6493
Patient 347	0/1	2038.6255
Patient 348	0/1	1540.5079
Patient 349	0/1	2726.68
Patient 350	0/1	2312.6886
Patient 351	0/1	5697.6444
Patient 352	0/1	806.3233
Patient 353	0/1	7956.4616
Patient 354	0/1	304.749
Patient 355	0/1	5788.4669
Patient 356	0/1	355.8132
Patient 357	0/1	496.729
Patient 358	0/1	2046.7591
Patient 359	0/1	4532.3178
Patient 360	0/1	280.1132
Patient 361	0/1	4246.804
Patient 362	0/1	2437.7524
Patient 363	0/1	397.3655
Patient 364	0/1	3776.6648
Patient 365	0/1	2346.5642
Patient 366	0/1	225.6105
Patient 367	0/1	1152.6834
Patient 368	0/1	1536.8006
Patient 369	0/1	338.7293

Patient 370	0/1	2980.8977
Patient 371	0/1	1797.5116
Patient 372	0/1	2771.3115
Patient 373	0/1	3118.1019
Patient 374	0/1	2852.4674
Patient 375	0/1	514.4949
Patient 376	0/1	1904.8943
Patient 377	0/1	4310.4
Patient 378	0/1	270.7385
Patient 379	0/1	5731.9914
Patient 380	1/1	723.5719
Patient 381	1/1	3989.2381
Patient 382	1/1	3281.434
Patient 383	1/1	1746.1051
Patient 384	1/1	1232.6139
Patient 385	1/1	538.6895
Patient 386	1/1	1232.9383
Patient 387	1/1	7875.7196
Patient 388	1/1	2255.8513
Patient 389	1/1	1425.9862
Patient 390	1/1	3375.0332
Patient 391	1/1	969.5481
Patient 392	1/1	1237.917
Patient 393	1/1	871.9662
Patient 394	1/1	2216.5064
Patient 395	1/1	522.6402
Patient 396	1/1	5036.8535
Patient 397	1/1	2090.4901
Patient 398	1/1	1716.1197
Patient 399	1/1	1632.3403
Patient 400	1/1	3487.2904
Patient 401	1/1	1938.755
Patient 402	1/1	1101.9725
Patient 403	1/1	518.1321
Patient 404	1/1	2553.7408
Patient 405	1/1	2009.3181
Patient 406	1/1	3015.2057
Patient 407	1/1	3198.6063
Patient 408	1/1	608.2916
Patient 409	1/1	1213.1703
Patient 410	1/1	5290.8668
Patient 411	1/1	735.7676
Patient 412	1/1	774.1749
Patient 413	1/1	1595.706
Patient 414	1/1	1222.0497
Patient 415	1/1	1761.6388
Patient 416	1/1	1214.1381
Patient 417	1/1	2517.2678
Patient 418	1/1	2510.4509
Patient 419	1/1	773.3564
Patient 420	1/1	797.4268
Patient 421	1/1	2140.0508
Patient 422	1/1	4695.2293

Patient 423	1/1	1454.2125
Patient 424	1/1	436.3188
Patient 425	1/1	4683.8818
Patient 426	1/1	2253.5679
Patient 427	1/1	759.6413
Patient 428	1/1	3770.2925
Patient 429	1/1	1891.3481
Patient 430	1/1	1232.4855
Patient 431	1/1	2144.101
Patient 432	1/1	1996.4934
Patient 433	1/1	3199.2379
Patient 434	1/1	2271.7611

	Colon Adenocarcinoma	
Patient ID	BCL2 Genotype	BCL2 normalized_count
Patient 1	0/0	108.9077
Patient 2	0/0	148.0916
Patient 3	0/0	221.5896
Patient 4	0/0	95.5723
Patient 5	0/0	42.1459
Patient 6	0/0	106.6667
Patient 7	0/0	242.0727
Patient 8	0/0	196.7905
Patient 9	0/0	99.2647
Patient 10	0/0	81.6075
Patient 11	0/0	256.5348
Patient 12	0/0	67.5547
Patient 13	0/0	25
Patient 14	0/0	269.802
Patient 15	0/0	32.2034
Patient 16	0/0	87.2618
Patient 17	0/0	31.4385
Patient 18	0/0	75.4323
Patient 19	0/1	66.5188
Patient 20	0/1	38.6965
Patient 21	0/1	218.6495
Patient 22	0/1	68.5579
Patient 23	0/1	156.6986
Patient 24	0/1	149.2537
Patient 25	0/1	247.3684
Patient 26	0/1	92.1659
Patient 27	0/1	83.887
Patient 28	0/1	91.629
Patient 29	0/1	213.8327
Patient 30	0/1	145.3287
Patient 31	0/1	35.489
Patient 32	0/1	375
Patient 33	0/1	45.601
Patient 34	0/1	122.807
Patient 35	0/1	85.3971
Patient 36	0/1	189.0587
Patient 37	0/1	44.5183
Patient 38	0/1	274.1935
Patient 39	0/1	78.668
Patient 40	0/1	470.8405
Patient 41	0/1	104.6632
Patient 42	0/1	223.0539
Patient 43	1/1	44.0068
Patient 44	1/1	564.2113
Patient 45	1/1	61.8446
Patient 46	1/1	347.0887
Patient 47	1/1	181.772
Patient 48	1/1	300
Patient 49	1/1	243.6054
Patient 50	1/1	94.2978

Uterine Corpus Endometrial Carcinoma		
Patient ID	BCL2 Genotype	BCL2 normalized_count
Patient 1	0/0	12.2137
Patient 2	0/0	30.7692
Patient 3	0/0	32.9457
Patient 4	0/0	41.2017
Patient 5	0/0	47.4061
Patient 6	0/0	48.1362
Patient 7	0/0	76.9231
Patient 8	0/0	80.0988
Patient 9	0/0	83.5808
Patient 10	0/0	91.1602
Patient 11	0/0	92.161
Patient 12	0/0	100.5803
Patient 13	0/0	102.8522
Patient 14	0/0	114.7059
Patient 15	0/0	116.8353
Patient 16	0/0	117.4986
Patient 17	0/0	122.2767
Patient 18	0/0	131.4508
Patient 19	0/0	134.8051
Patient 20	0/0	139.3981
Patient 21	0/0	141.0756
Patient 22	0/0	142.171
Patient 23	0/0	145.0382
Patient 24	0/0	146.0374
Patient 25	0/0	149.9898
Patient 26	0/0	151.0216
Patient 27	0/0	152.5206
Patient 28	0/0	152.7016
Patient 29	0/0	156.5605
Patient 30	0/0	158.0189
Patient 31	0/0	159.497
Patient 32	0/0	159.7281
Patient 33	0/0	164.4295
Patient 34	0/0	166.2588
Patient 35	0/0	171.4155
Patient 36	0/0	175.0663
Patient 37	0/0	181.0251
Patient 38	0/0	181.2373
Patient 39	0/0	182.598
Patient 40	0/0	185.4839
Patient 41	0/0	194.4419
Patient 42	0/0	194.6523
Patient 43	0/0	196.7551
Patient 44	0/0	198.8218
Patient 45	0/0	207.0626
Patient 46	0/0	207.2072
Patient 47	0/0	210.0947
Patient 48	0/0	211.4286
Patient 49	0/0	222.4035
Patient 50	0/0	223.1134
Patient 51	0/0	251.4549

Patient 52	0/0	258.5513
Patient 53	0/0	260.6122
Patient 54	0/0	264.3794
Patient 55	0/0	276.7296
Patient 56	0/0	290.5983
Patient 57	0/0	293.6508
Patient 58	0/0	301.2884
Patient 59	0/0	302.8217
Patient 60	0/0	307.6923
Patient 61	0/0	314.1732
Patient 62	0/0	321.8274
Patient 63	0/0	324.9243
Patient 64	0/0	334.124
Patient 65	0/0	334.4393
Patient 66	0/0	342.9188
Patient 67	0/0	345.2514
Patient 68	0/0	346.0765
Patient 69	0/0	361.4697
Patient 70	0/0	362.7119
Patient 71	0/0	374.6436
Patient 72	0/0	376.2201
Patient 73	0/0	386.0182
Patient 74	0/0	388.6384
Patient 75	0/0	388.705
Patient 76	0/0	399.1736
Patient 77	0/0	409.8876
Patient 78	0/0	426.3566
Patient 79	0/0	429.5175
Patient 80	0/0	442.5703
Patient 81	0/0	463.0886
Patient 82	0/0	464.4809
Patient 83	0/0	474.8092
Patient 84	0/0	505.4825
Patient 85	0/0	508.3889
Patient 86	0/0	522.6205
Patient 87	0/0	524.5642
Patient 88	0/0	544.7059
Patient 89	0/0	553.7313
Patient 90	0/0	558.9471
Patient 91	0/0	597.551
Patient 92	0/0	612.8266
Patient 93	0/0	645.3243
Patient 94	0/0	664.5265
Patient 95	0/0	693.6566
Patient 96	0/0	760.4252
Patient 97	0/0	761.2775
Patient 98	0/0	768.2841
Patient 99	0/0	830.3078
Patient 100	0/0	834.8624
Patient 101	0/0	874.0895
Patient 102	0/0	882.0034
Patient 103	0/0	1026.0536
Patient 104	0/0	1074.5131

Patient 105	0/0	1091.3295
Patient 106	0/0	1099.4764
Patient 107	0/0	1126.9406
Patient 108	0/0	1181.3785
Patient 109	0/0	1204.0486
Patient 110	0/1	9.0016
Patient 111	0/1	21.5558
Patient 112	0/1	33.6134
Patient 113	0/1	71.9603
Patient 114	0/1	85.3549
Patient 115	0/1	100.6711
Patient 116	0/1	101.5435
Patient 117	0/1	118.0822
Patient 118	0/1	127.907
Patient 119	0/1	131.1475
Patient 120	0/1	139.6855
Patient 121	0/1	144.972
Patient 122	0/1	145.8094
Patient 123	0/1	149.8439
Patient 124	0/1	150.0469
Patient 125	0/1	155.9858
Patient 126	0/1	157.2917
Patient 127	0/1	160.555
Patient 128	0/1	169.9422
Patient 129	0/1	177.8127
Patient 130	0/1	183.6066
Patient 131	0/1	186.2606
Patient 132	0/1	197.0199
Patient 133	0/1	199.6916
Patient 134	0/1	203.9882
Patient 135	0/1	212.2905
Patient 136	0/1	215.2659
Patient 137	0/1	218.0268
Patient 138	0/1	225.8953
Patient 139	0/1	226.3682
Patient 140	0/1	232.9337
Patient 141	0/1	234.3298
Patient 142	0/1	240.2707
Patient 143	0/1	243.787
Patient 144	0/1	248.1061
Patient 145	0/1	249.3579
Patient 146	0/1	250.8718
Patient 147	0/1	259.5081
Patient 148	0/1	260.1344
Patient 149	0/1	284.9604
Patient 150	0/1	287.0293
Patient 151	0/1	302.0408
Patient 152	0/1	314.2523
Patient 153	0/1	314.3759
Patient 154	0/1	317.6692
Patient 155	0/1	320.7721
Patient 156	0/1	325.2496
Patient 157	0/1	336.3983

Patient 158	0/1	337.4517
Patient 159	0/1	348.964
Patient 160	0/1	352.4069
Patient 161	0/1	354.1102
Patient 162	0/1	358.9722
Patient 163	0/1	395.0497
Patient 164	0/1	398.4018
Patient 165	0/1	400.2478
Patient 166	0/1	405.0926
Patient 167	0/1	423.0453
Patient 168	0/1	433.9051
Patient 169	0/1	444.2211
Patient 170	0/1	455.7274
Patient 171	0/1	459.6555
Patient 172	0/1	474.122
Patient 173	0/1	474.2268
Patient 174	0/1	476.8911
Patient 175	0/1	479.6031
Patient 176	0/1	508.7282
Patient 177	0/1	514.4764
Patient 178	0/1	515.4062
Patient 179	0/1	533.0113
Patient 180	0/1	541.9103
Patient 181	0/1	545.2128
Patient 182	0/1	559.9255
Patient 183	0/1	560.1907
Patient 184	0/1	571.8424
Patient 185	0/1	583.7054
Patient 186	0/1	621.3115
Patient 187	0/1	632.613
Patient 188	0/1	644.7587
Patient 189	0/1	666.8425
Patient 190	0/1	730.5503
Patient 191	0/1	760.8491
Patient 192	0/1	779.0507
Patient 193	0/1	793.5256
Patient 194	0/1	796.6616
Patient 195	0/1	849.3447
Patient 196	0/1	864.0107
Patient 197	0/1	865.2947
Patient 198	0/1	967.6674
Patient 199	0/1	1063.4706
Patient 200	0/1	1092.7786
Patient 201	0/1	1138.1974
Patient 202	0/1	1274.6694
Patient 203	0/1	1432.2034
Patient 204	0/1	1783.591
Patient 205	0/1	1999.0282
Patient 206	0/1	2383.3866
Patient 207	0/1	471.9711
Patient 208	1/1	17.2228
Patient 209	1/1	63.5386
Patient 210	1/1	64.6614

Patient 211	1/1	99.1836
Patient 212	1/1	118.5243
Patient 213	1/1	134.5219
Patient 214	1/1	156.8627
Patient 215	1/1	171.1957
Patient 216	1/1	186.1979
Patient 217	1/1	214.9787
Patient 218	1/1	237.8698
Patient 219	1/1	251.4718
Patient 220	1/1	262.0522
Patient 221	1/1	275.0929
Patient 222	1/1	302.866
Patient 223	1/1	310.7948
Patient 224	1/1	352.0209
Patient 225	1/1	359.8485
Patient 226	1/1	449.2923
Patient 227	1/1	499.6091
Patient 228	1/1	564.6867
Patient 229	1/1	564.7284
Patient 230	1/1	572.4044
Patient 231	1/1	618.3144
Patient 232	1/1	640.6571
Patient 233	1/1	689.6552
Patient 234	1/1	744.8985
Patient 235	1/1	818.436
Patient 236	1/1	966.9065
Patient 237	1/1	1039.3542
Patient 238	1/1	1258.6207
Patient 239	1/1	1280.9524
Patient 240	1/1	1394.5312
Patient 241	1/1	1563.4409

Head and Neck squamous cell carcinoma

Patient ID	BCL2 Genotype	BCL2 normalized_count
Patient 1	0/0	23.6128
Patient 2	0/0	57.4949
Patient 3	0/0	138.5779
Patient 4	0/0	212.5506
Patient 5	0/0	31.796
Patient 6	0/0	95.1715
Patient 7	0/0	315.6207
Patient 8	0/0	36.4474
Patient 9	0/0	531.8904
Patient 10	0/0	101.6284
Patient 11	0/0	242.1454
Patient 12	0/0	272.8152
Patient 13	0/0	99.627
Patient 14	0/0	68.3325
Patient 15	0/0	179.0795
Patient 16	0/0	177.682
Patient 17	0/0	396.7631
Patient 18	0/0	177.6661
Patient 19	0/0	158.8889
Patient 20	0/0	290.7276
Patient 21	0/0	91.1728
Patient 22	0/0	179.3339
Patient 23	0/0	30.5316
Patient 24	0/0	170.9522
Patient 25	0/0	147.1742
Patient 26	0/0	73.6352
Patient 27	0/0	60.5624
Patient 28	0/0	233.3557
Patient 29	0/0	191.7071
Patient 30	0/0	163.3825
Patient 31	0/0	109.2009
Patient 32	0/0	796.3025
Patient 33	0/0	74.4625
Patient 34	0/0	555.0595
Patient 35	0/0	599.5996
Patient 36	0/0	260.7616
Patient 37	0/0	64.2101
Patient 38	0/0	40.4002
Patient 39	0/0	347.8517
Patient 40	0/0	195.0133
Patient 41	0/0	282.3872
Patient 42	0/0	802.9872
Patient 43	0/0	151.1389
Patient 44	0/0	85.0508
Patient 45	0/0	288.8289
Patient 46	0/0	339.9193
Patient 47	0/0	168.6748
Patient 48	0/0	442.0154
Patient 49	0/0	181.6879
Patient 50	0/0	377.3032
Patient 51	0/0	351.253

Patient 52	0/0	413.451
Patient 53	0/0	123.6946
Patient 54	0/0	467.5135
Patient 55	0/0	275.5
Patient 56	0/0	75.5208
Patient 57	0/0	444.4444
Patient 58	0/0	23.5384
Patient 59	0/0	197.3606
Patient 60	0/0	45.5655
Patient 61	0/0	92.8583
Patient 62	0/0	86.4562
Patient 63	0/0	130.201
Patient 64	0/0	159.4985
Patient 65	0/0	109.8926
Patient 66	0/0	226.6667
Patient 67	0/0	169.5749
Patient 68	0/0	238.8693
Patient 69	0/0	127.8477
Patient 70	0/0	125.4094
Patient 71	0/0	153.7525
Patient 72	0/0	488.7431
Patient 73	0/0	72.8583
Patient 74	0/0	237.9928
Patient 75	0/0	186.4917
Patient 76	0/0	18.3095
Patient 77	0/0	191.2582
Patient 78	0/0	108.9267
Patient 79	0/0	297.136
Patient 80	0/0	365.7961
Patient 81	0/0	29.2869
Patient 82	0/0	181.3222
Patient 83	0/0	243.2122
Patient 84	0/0	154.9296
Patient 85	0/0	747.1984
Patient 86	0/0	388.735
Patient 87	0/0	94.2847
Patient 88	0/0	124.6386
Patient 89	0/0	82.786
Patient 90	0/0	228.4925
Patient 91	0/0	176.2846
Patient 92	0/0	154.8878
Patient 93	0/0	67.6799
Patient 94	0/0	27.9572
Patient 95	0/0	205.8957
Patient 96	0/0	57.8356
Patient 97	0/0	62.4342
Patient 98	0/0	81.0515
Patient 99	0/0	36.855
Patient 100	0/0	89.0566
Patient 101	0/0	496.7051
Patient 102	0/0	134.1648
Patient 103	0/1	47.7873
Patient 104	0/1	117.4206

Patient 105	0/1	1050.6984
Patient 106	0/1	226.2842
Patient 107	0/1	312.1163
Patient 108	0/1	714.3441
Patient 109	0/1	80.3493
Patient 110	0/1	187.3568
Patient 111	0/1	1446.4496
Patient 112	0/1	93.8101
Patient 113	0/1	73.6339
Patient 114	0/1	118.3729
Patient 115	0/1	115.7943
Patient 116	0/1	368.3951
Patient 117	0/1	178.1999
Patient 118	0/1	159.5952
Patient 119	0/1	91.1024
Patient 120	0/1	402.9787
Patient 121	0/1	1414.4284
Patient 122	0/1	83.6302
Patient 123	0/1	45.6048
Patient 124	0/1	113.8702
Patient 125	0/1	242.8965
Patient 126	0/1	46.538
Patient 127	0/1	292.6254
Patient 128	0/1	238.1198
Patient 129	0/1	441.3292
Patient 130	0/1	111.0815
Patient 131	0/1	392.1635
Patient 132	0/1	81.0337
Patient 133	0/1	912.4706
Patient 134	0/1	250.1587
Patient 135	0/1	315.7284
Patient 136	0/1	132.7112
Patient 137	0/1	303.3782
Patient 138	0/1	201.0078
Patient 139	0/1	89.6781
Patient 140	0/1	73.5931
Patient 141	0/1	209.3023
Patient 142	0/1	299.3859
Patient 143	0/1	139.4953
Patient 144	0/1	352.2592
Patient 145	0/1	152.4709
Patient 146	0/1	479.2379
Patient 147	0/1	2187.6697
Patient 148	0/1	155.8484
Patient 149	0/1	148.9511
Patient 150	0/1	235.9045
Patient 151	0/1	874.3828
Patient 152	0/1	173.9444
Patient 153	0/1	122.28
Patient 154	0/1	192.8971
Patient 155	0/1	448.0052
Patient 156	0/1	82.7754
Patient 157	0/1	291.8306

Patient 158	0/1	70.3902
Patient 159	0/1	273.9461
Patient 160	0/1	290.827
Patient 161	0/1	179.0614
Patient 162	0/1	237.798
Patient 163	0/1	670.5272
Patient 164	0/1	59.0823
Patient 165	0/1	884.1535
Patient 166	0/1	31.8302
Patient 167	0/1	44.288
Patient 168	0/1	25.9924
Patient 169	0/1	1089.0599
Patient 170	0/1	49.355
Patient 171	0/1	378.9765
Patient 172	0/1	136.9716
Patient 173	0/1	58.5963
Patient 174	0/1	133.3846
Patient 175	0/1	94.1029
Patient 176	0/1	185.4542
Patient 177	0/1	255.2248
Patient 178	0/1	157.824
Patient 179	0/1	113.485
Patient 180	0/1	88.703
Patient 181	0/1	137.3874
Patient 182	0/1	77.1717
Patient 183	0/1	104.9618
Patient 184	0/1	102.9134
Patient 185	0/1	554.2582
Patient 186	0/1	210.0207
Patient 187	0/1	98.9207
Patient 188	0/1	283.5109
Patient 189	0/1	28.3727
Patient 190	0/1	360.5214
Patient 191	0/1	77.2947
Patient 192	0/1	203.6954
Patient 193	0/1	72.5029
Patient 194	0/1	411.1028
Patient 195	0/1	38.4763
Patient 196	0/1	99.5738
Patient 197	0/1	80.8752
Patient 198	0/1	99.2781
Patient 199	0/1	315.264
Patient 200	0/1	296.4918
Patient 201	0/1	127.6117
Patient 202	0/1	115.4522
Patient 203	0/1	3849.4726
Patient 204	0/1	313.0983
Patient 205	0/1	41.0159
Patient 206	0/1	31.4301
Patient 207	0/1	248.1023
Patient 208	1/1	2236.3775
Patient 209	1/1	2658.0372
Patient 210	1/1	83.1306

Patient 211	1/1	2682.1393
Patient 212	1/1	2401.4343
Patient 213	1/1	49.175
Patient 214	1/1	175.5551
Patient 215	1/1	245.4338
Patient 216	1/1	228.1713
Patient 217	1/1	173.9427
Patient 218	1/1	196.9843
Patient 219	1/1	463.2116
Patient 220	1/1	183.6575
Patient 221	1/1	273.8666
Patient 222	1/1	43.5075
Patient 223	1/1	194.5312
Patient 224	1/1	314.996
Patient 225	1/1	352.4385
Patient 226	1/1	852.4043
Patient 227	1/1	464.2772
Patient 228	1/1	180.5237
Patient 229	1/1	49.4202
Patient 230	1/1	105.2061
Patient 231	1/1	48.2436
Patient 232	1/1	33.8983
Patient 233	1/1	52.6316
Patient 234	1/1	166.0996
Patient 235	1/1	44.5205
Patient 236	1/1	78.6385
Patient 237	1/1	58.5982
Patient 238	1/1	1515.765
Patient 239	1/1	121.0215
Patient 240	1/1	104.7103
Patient 241	1/1	81.0903
Patient 242	1/1	67.0227
Patient 243	1/1	375.0994
Patient 244	1/1	146.6724

Patient ID	Kidney Chromophobe BCL2 Genotype	BCL2 normalized_count
Patient 1	0/0	786.135
Patient 2	0/0	758.6668
Patient 3	0/0	1706.2628
Patient 4	0/0	2304.584
Patient 5	0/0	2281.7206
Patient 6	0/0	99.6057
Patient 7	0/0	1347.8743
Patient 8	0/0	2486.7456
Patient 9	0/0	856.3574
Patient 10	0/0	1899.6639
Patient 11	0/0	1900.1068
Patient 12	0/0	2217.4515
Patient 13	0/0	1886.0234
Patient 14	0/0	2612.5739
Patient 15	0/0	2428.2914
Patient 16	0/0	2180.7989
Patient 17	0/0	2658.43
Patient 18	0/0	1702.7273
Patient 19	0/1	2627.3496
Patient 20	0/1	2409.0739
Patient 21	0/1	1967.0504
Patient 22	0/1	2156.7444
Patient 23	0/1	779.6053
Patient 24	0/1	3584.7211
Patient 25	0/1	2731.7179
Patient 26	0/1	2505.4657
Patient 27	0/1	1626.0658
Patient 28	0/1	2730.6016
Patient 29	0/1	911.3631
Patient 30	0/1	3737.3567
Patient 31	0/1	3001.3471
Patient 32	0/1	2054.0362
Patient 33	0/1	715.7623
Patient 34	0/1	1506.713
Patient 35	0/1	2428.8151
Patient 36	0/1	3737.9982
Patient 37	0/1	1249.0354
Patient 38	0/1	3160.3774
Patient 39	0/1	899.6721
Patient 40	1/1	3006.1359
Patient 41	1/1	2989.1734
Patient 42	1/1	3825.0708
Patient 43	1/1	1811.3821
Patient 44	1/1	1592.6875
Patient 45	1/1	3383.2414
Patient 46	1/1	1245.3988
Patient 47	1/1	2159.6226
Patient 48	1/1	3026.2748
Patient 49	1/1	1108.5367
Patient 50	1/1	2079.8872
Patient 51	1/1	2285.6003

Patient 52	1/1	2209.3788
Patient 53	1/1	3148.855
Patient 54	1/1	1449.0174
Patient 55	1/1	1316.8103
Patient 56	1/1	1556.2585

	Thyroid Carcinoma	
Patient ID	BCL2 Genotype	BCL2 normalized_count
Patient 1	0/0	521.8411
Patient 2	0/0	611.805
Patient 3	0/0	1275.1092
Patient 4	0/0	618.9125
Patient 5	0/0	2104.9563
Patient 6	0/0	926.3127
Patient 7	0/0	1142.0505
Patient 8	0/0	2165.8463
Patient 9	0/0	2041.6139
Patient 10	0/0	1509.3108
Patient 11	0/0	1817.9262
Patient 12	0/0	559.0062
Patient 13	0/0	1115.4609
Patient 14	0/0	800.8822
Patient 15	0/0	835.6654
Patient 16	0/0	1281.4269
Patient 17	0/0	875.3139
Patient 18	0/0	922.4137
Patient 19	0/0	996.0044
Patient 20	0/0	1397.4503
Patient 21	0/0	824.365
Patient 22	0/0	1745.2305
Patient 23	0/0	889.771
Patient 24	0/0	503.6496
Patient 25	0/0	671.7511
Patient 26	0/0	1072.9443
Patient 27	0/0	1160.181
Patient 28	0/0	1322.0928
Patient 29	0/0	658.5239
Patient 30	0/0	934.7518
Patient 31	0/0	734.6165
Patient 32	0/0	916
Patient 33	0/0	425.1831
Patient 34	0/0	1257.7154
Patient 35	0/0	662.7994
Patient 36	0/0	3238.5374
Patient 37	0/0	3811.5265
Patient 38	0/0	1621.1343
Patient 39	0/0	466.4213
Patient 40	0/0	1505.772
Patient 41	0/0	1232.493
Patient 42	0/0	1303.909
Patient 43	0/0	3908.5516
Patient 44	0/0	888.3014
Patient 45	0/0	455.7395
Patient 46	0/0	730.6074
Patient 47	0/0	978.6535
Patient 48	0/0	1093.1152
Patient 49	0/0	1133.7053
Patient 50	0/0	736.548
Patient 51	0/0	1858.363

Patient 52	0/0	1172.685
Patient 53	0/0	740.7913
Patient 54	0/0	790.6538
Patient 55	0/0	1072.2541
Patient 56	0/0	956.1827
Patient 57	0/0	674.7619
Patient 58	0/0	1635.152
Patient 59	0/0	1248.5918
Patient 60	0/0	510.8866
Patient 61	0/0	773.4316
Patient 62	0/0	1394.9612
Patient 63	0/0	1313.0207
Patient 64	0/0	568.3591
Patient 65	0/0	866.3594
Patient 66	0/0	626.0627
Patient 67	0/0	1066.8419
Patient 68	0/0	762.8885
Patient 69	0/0	727.963
Patient 70	0/0	777.8995
Patient 71	0/0	935.395
Patient 72	0/0	837.9236
Patient 73	0/0	1668.0517
Patient 74	0/0	1181.3472
Patient 75	0/0	2406.168
Patient 76	0/0	1289.3993
Patient 77	0/0	765.0688
Patient 78	0/0	633.8922
Patient 79	0/0	660.1791
Patient 80	0/0	300.4585
Patient 81	0/1	1077.0314
Patient 82	0/1	1449.3146
Patient 83	0/1	3880.597
Patient 84	0/1	2473.6472
Patient 85	0/1	1276.5305
Patient 86	0/1	2210.0562
Patient 87	0/1	855.506
Patient 88	0/1	572.9948
Patient 89	0/1	1035.4403
Patient 90	0/1	1147.244
Patient 91	0/1	2075.5813
Patient 92	0/1	982.7262
Patient 93	0/1	438.3726
Patient 94	0/1	194.8718
Patient 95	0/1	377.9621
Patient 96	0/1	1395.6743
Patient 97	0/1	4974.3685
Patient 98	0/1	1146.395
Patient 99	0/1	813.8764
Patient 100	0/1	4194.2446
Patient 101	0/1	2049.9024
Patient 102	0/1	3142.8964
Patient 103	0/1	1748.841
Patient 104	0/1	3159.8768

Patient 105	0/1	1520.6443
Patient 106	0/1	2838.8052
Patient 107	0/1	746.1838
Patient 108	0/1	708.9128
Patient 109	0/1	1558.3864
Patient 110	0/1	5345.1327
Patient 111	0/1	2033.4957
Patient 112	0/1	329.407
Patient 113	0/1	1230.9942
Patient 114	0/1	638.7833
Patient 115	0/1	616.7104
Patient 116	0/1	3830.7275
Patient 117	0/1	583.5174
Patient 118	0/1	3233.3471
Patient 119	0/1	1679.0222
Patient 120	0/1	671.5718
Patient 121	0/1	1146.8095
Patient 122	0/1	1026.1289
Patient 123	0/1	610.4486
Patient 124	0/1	3261.5977
Patient 125	0/1	3650.6159
Patient 126	0/1	753.2963
Patient 127	0/1	980.5556
Patient 128	0/1	364.4702
Patient 129	0/1	725.8602
Patient 130	0/1	5229.5875
Patient 131	0/1	2373.4919
Patient 132	0/1	906.8001
Patient 133	0/1	293.6225
Patient 134	0/1	653.1489
Patient 135	0/1	992.2898
Patient 136	0/1	891.8744
Patient 137	0/1	531.5722
Patient 138	0/1	2447.6744
Patient 139	0/1	491.5949
Patient 140	0/1	792.3619
Patient 141	0/1	1064.0184
Patient 142	0/1	1107.0533
Patient 143	0/1	166.0478
Patient 144	0/1	430.9327
Patient 145	0/1	440.6919
Patient 146	0/1	2172.6519
Patient 147	0/1	2210.0251
Patient 148	0/1	790.7508
Patient 149	0/1	1220.5376
Patient 150	0/1	2196.323
Patient 151	0/1	564.2523
Patient 152	0/1	630.6272
Patient 153	0/1	1179.1159
Patient 154	0/1	1498.4967
Patient 155	0/1	659.5112
Patient 156	0/1	795.8861
Patient 157	0/1	2647.5431

Patient 158	0/1	2431.8477
Patient 159	0/1	863.5169
Patient 160	0/1	1061.5438
Patient 161	0/1	363.0205
Patient 162	0/1	828.9916
Patient 163	0/1	1337.2549
Patient 164	0/1	2505.4367
Patient 165	0/1	1101.1879
Patient 166	0/1	355.8809
Patient 167	0/1	1437.454
Patient 168	0/1	679.95
Patient 169	0/1	198.8764
Patient 170	0/1	154.5059
Patient 171	0/1	440.2108
Patient 172	0/1	436.409
Patient 173	0/1	3055.5799
Patient 174	0/1	499.6583
Patient 175	0/1	1170.2404
Patient 176	0/1	895.2726
Patient 177	0/1	4743.4515
Patient 178	0/1	1198.324
Patient 179	0/1	647.1031
Patient 180	0/1	1838.3574
Patient 181	0/1	1174.3606
Patient 182	0/1	1254.5113
Patient 183	0/1	946.3616
Patient 184	0/1	2208.6858
Patient 185	0/1	1262.0798
Patient 186	0/1	1343.6599
Patient 187	0/1	1157.2728
Patient 188	0/1	916.6667
Patient 189	1/1	2386.9969
Patient 190	1/1	1644.2235
Patient 191	1/1	233.2941
Patient 192	1/1	475.0733
Patient 193	1/1	475.8906
Patient 194	1/1	1032.4943
Patient 195	1/1	2195.0207
Patient 196	1/1	2518.5328
Patient 197	1/1	378.7301
Patient 198	1/1	2054.4904
Patient 199	1/1	1079.6445
Patient 200	1/1	898.4005
Patient 201	1/1	854.3486
Patient 202	1/1	3299.8963
Patient 203	1/1	913.4232
Patient 204	1/1	1047.9476
Patient 205	1/1	324.7974
Patient 206	1/1	922.6463
Patient 207	1/1	743.0453
Patient 208	1/1	1660.4451
Patient 209	1/1	3098.5435
Patient 210	1/1	1210.5464

Patient 211	1/1	2336.1796
Patient 212	1/1	2379.1304
Patient 213	1/1	1227.2653
Patient 214	1/1	958.3333
Patient 215	1/1	640.5312
Patient 216	1/1	583.6444
Patient 217	1/1	5324.7275
Patient 218	1/1	698.4646
Patient 219	1/1	916.2037
Patient 220	1/1	1326.6917
Patient 221	1/1	1227.0512
Patient 222	1/1	551.5051
Patient 223	1/1	468.9716
Patient 224	1/1	1230.4772
Patient 225	1/1	531.6775
Patient 226	1/1	605.9841
Patient 227	1/1	1876.087
Patient 228	1/1	839.1204
Patient 229	1/1	4381.9424
Patient 230	1/1	2981.502
Patient 231	1/1	383.4478
Patient 232	1/1	1605.7723
Patient 233	1/1	1339.3991
Patient 234	1/1	550.4556
Patient 235	1/1	2008.8889
Patient 236	1/1	870.7533
